# Derivatives of the Cashew Nut Shell Liquid as Ligands of *Pseudomonas aeruginosa* Phenazine Protein D (*Pa*PhzD)

**DOI:** 10.1021/acsomega.5c08941

**Published:** 2025-12-15

**Authors:** Marina Sena Mendes, Thamires Quadros Froes, Caio Gomes Tavares Rosa, Gabriella Simões Heyn Roth Cardoso, Thais Ferreira, Andressa Souza de Oliveira, Luiz A. S. Romeiro, Regina Lúcia Baldini, Marcelo S. Castilho

**Affiliations:** † 495454 Faculty of Pharmacy, Federal University of Bahia (UFBA), 40140-115 Salvador, Bahia, Brazil; ‡ Instituto de Pesquisa Gonçalo Moniz—Fiocruz, Bahia 40296-710, Brazil; § Programa de Pós-graduação em Ciências Farmacêuticas, Faculdade de Ciências da Saúde, UnB, Brasilia 70000-000, Brazil; ∥ Programa de Pós-graduação em Medicina Tropical, Faculdade de Medicina, UnB, Brasilia 70910-900, Brazil; ⊥ Departamento de Bioquímica, Instituto de Química, 28133 Universidade de São Paulo, 05508-900 Sao Paulo, Brazil

## Abstract

*Pseudomonas aeruginosa* isochorismatase PhzD (*Pa*PhzD) is key to the biosynthesis of pyocyanin (PYO), a virulence factor facilitating pathogen infection and survival within the host. To date, no ligands of *Pa*PhzD have been reported. Leveraging the chemical similarity between anacardic acid derivatives and the enzyme’s natural substrate, three compounds (2, 3, and 4) were identified as low to submicromolar *Pa*PhzD ligands, while compounds 2 and 4 reduced pyocyanin production (Cohen′d = −2.56 and −2.69, respectively) and swarming motility (Cohen′d = −0.91 and −0.95, respectively) at 100 μM, without affecting bacterial growth or biofilm formation (*p* > 0.05). Our results suggest that compounds binding to *Pa*PhzD or PPAR have distinct SAR requirements, suggesting that they represent a promising scaffold for developing *Pa*PhzD inhibitors.

## Graphical abstract

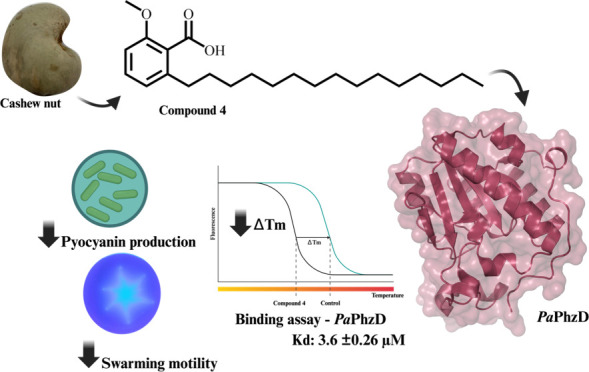

## 1 Introduction

Antimicrobial resistance (AMR) is a worldwide threat to public health due to the high morbidity and mortality from infections, for which there is no effective treatment available. As a result, approximately 35,000 individuals die each year in Europe from infectious diseases caused by drug-resistant bacteria,^1^ while 214,000 newborns lose their lives to AMR in low- and middle-income countries per year.^2^ Apart from the huge impact on human lives, AMR also claims its toll on healthcare systems.^3^ Despite this scenario, the investment of pharmaceutical companies in antibiotic development has been decreasing over the past few decades.^4,5^ Since the 1970s, four novel classes of antibiotics have reached the market.^6^ Three of these target Gram-negative bacteria in a limited way.^7–9^ One reason for the lack of interest in antimicrobial drugs is that traditional strategies focus on developing bacteriostatic or bactericidal agents, which exert evolutionary pressure on microorganisms and, consequently, select resistant strains.^10^ To mitigate selection pressure, state-of-the-art drugs, which are developed at significant financial costs, are prescribed to only a limited number of patients. This renders antibacterial drug discovery a high-risk, low-profit endeavor.^11^

Comprehending the mechanisms of bacterial resistance and the immune system’s role in disease progression is of the utmost importance to overcome this situation.^12^ *Pseudomonas aeruginosa* is a ubiquitous bacterium that behaves as an opportunistic pathogen, infecting burns and surgical wounds, and grows as biofilms in catheters, prosthetic devices, and assisted ventilation apparatus, corresponding to one of the leading cases of hospital-acquired infection.^13–16^ During the COVID-19 pandemic, *P. aeruginosa* was frequently found coinfecting patients’ lungs, exacerbating respiratory symptoms.^17^ The high resistance of *P. aeruginosa* to antibiotics poses a challenge to treating infections caused by this bacterium, whose low outer membrane permeability, numerous efflux pumps, the expression of beta-lactamases, and horizontally acquired resistance genes add different layers to finding effective treatment,^18,19^ resulting in clinical isolates being resistant to all currently available antipseudomonal drugs.^20^

*P. aeruginosa* virulence depends on several factors,^21^ including proteases, siderophores, rhamnolipids, elastase, pyocyanin, and biofilm growth.^22^ Hence, decreasing bacterial virulence might be a promising strategy to fight infectious diseases.^23^

Among *P. aeruginosa* virulence factors, pyocyanin (PYO) has proinflammatory effects, causes a redox imbalance in the host cell,^23^ increases IL-8 and leukotriene B4 production within alveolar macrophages,^24^ thus resulting in cellular damage and death. Due to its biological relevance, PYO production inhibition has been exploited as a preliminary surrogate to select compounds that might have antivirulence activity.^25–28^

Natural products have been an invaluable source of bactericidal and bacteriostatic drugs,^29,30^ and their potential to control the virulence of *P. aeruginosa* is recognized.^31–34^ Our group described the effect of coumarin derivatives and calycopterin on *P. aeruginosa* motility, biofilm development, and PYO production.^35,36^ However, this work was based mainly on phenotypic assays that do not provide direct evidence of macromolecular target engagement. As several enzymes from the PYO biosynthesis pathway have been considered as druggable antivirulence targets,^37^ a structure-based approach was undertaken to identify inhibitors of *P. aeruginosa* PhzS (E. C. 1.14.13.218), a key enzyme in the PYO biosynthesis pathway.^35,38^

In our pursuit to identify versatile and renewable raw materials for designing sustainable and low-cost lead compounds, our research group has exploited nonisoprenoid phenolic lipids found in cashew (*Anacardium occidentale* L.) nut shells liquid (CNSL).^39^ The CNSL contains mixtures of anacardic acids, cardanols, cardols, and 2-methylcardols, featuring a 15-carbon alkyl chain that can be saturated or exhibit varying degrees of unsaturation as monoene, diene, or triene (Figure 1). These lipids, whether in their purified form or as mixtures, showcase diverse biological activities, including antioxidant, anti-inflammatory,^40^ antinociceptive,^41^ antimicrobial,^42,43^ and larvicidal^44^ properties. In this study, we report that derivatives of these compounds (LDT) also serve as low micromolar ligands for *P. aeruginosa* phenazine biosynthesis protein D (*Pa*PhzD) (E.C.3.3.2.15), an enzyme acting upstream of PhzS in the PYO biosynthesis pathway. Among the natural product derivatives evaluated, two (LDT **2** and **4**) bind to *Pa*PhzD, reducing PYO production and swarming motility without affecting bacterial growth, while a third compound (LDT **3**) reduces only biofilm formation. Preliminary structure–activity relationship (SAR) studies for this series indicate that a hydrogen bond acceptor, such as the acidic moiety found in anacardic acids or the phenolic OH present in cardanols, is essential for *Pa*PhzD binding.

**Figure 1:**
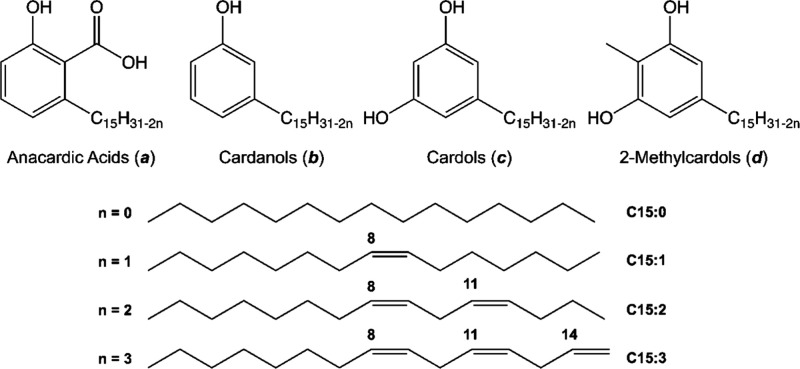
Phenolic lipids of the cashew nut shell liquid (CNSL).

## 2 Material and Methods

### 2.1 Chemistry Methods

The reagents and solvents employed were purchased from Sigma-Aldrich and used without further purification. The reactions were monitored by thin-layer chromatography (TLC) using Merck Silica gel 60 F254 chromatographic plates, 0.25 mm thick. All compounds were characterized by ^1^H and ^13^C NMR analyses (Supporting Information).

Compounds **1–16** (Figure 2) were obtained in good to moderate yields, as described previously.^45^ Briefly, anacardic acids (***a***) and cardanol (***b***) mixtures were obtained as described by Rossi et al.^46^ Hydrogenation of the unsaturated chains present in mixture *a* afforded compound **1**, from which compounds **3**, **4**, **7**, **8,** and **9** were obtained, as described previously.^46^ Compounds **5** and **6** were obtained from the reduction of compounds **1** and **9** with lithium aluminum hydride, providing the respective alcohols (Scheme 1). Catalytic hydrogenation of mixture *b* afforded compound **2**, which was transformed into salicylaldehyde **17**, converted to the methoxy derivative **18**, and then oxidized to 10. The cross-reference to the original LDT numbering employed in the previously published papers is shown in Table 1S.

**Scheme 1:**
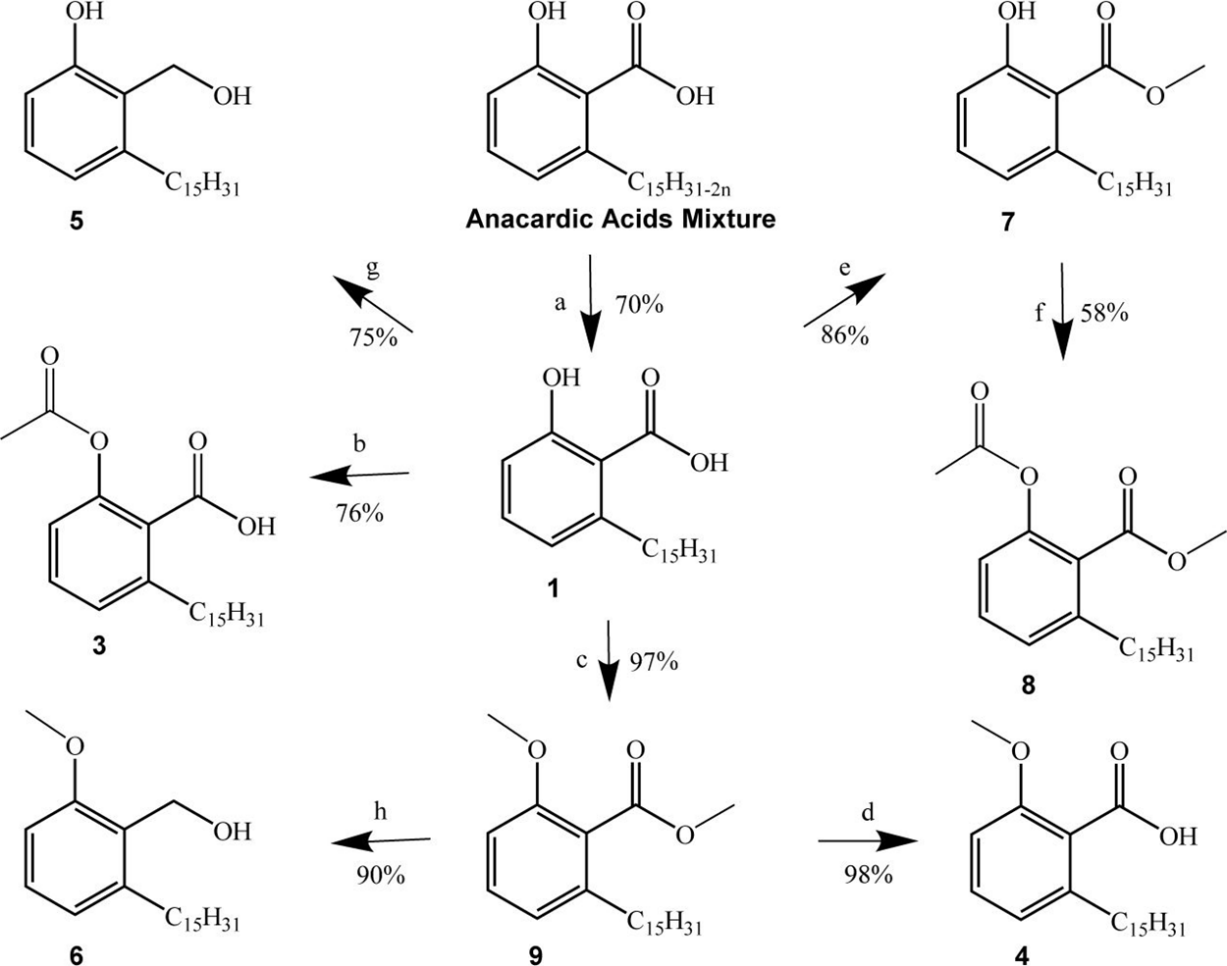
Synthesis of the Anacardic Acid Derivatives^a^

a Reagents and conditions: a. H_2,_ Pd/C 10%, EtOH, r.t., 6 h; b. AC_2_O, H_3_PO_4,_ MW, 3 min; c. MeI, K_2_CO_3,_ Me_2_CO, 120 °C, 16 h; d. *t*-BuOK, DMSO, 40 °C, 16 h; e. MeOH, H_2_SO_4,_ 50 °C, 16 h; f. AcCl, TEA, CH_2_Cl_2,_ r.t. Sixteen h; g. LiAlH_4_, THF, 66 °C, 18 h; h. LiAlH_4_, THF, 110 °C, 24 h.

**Figure 2:**
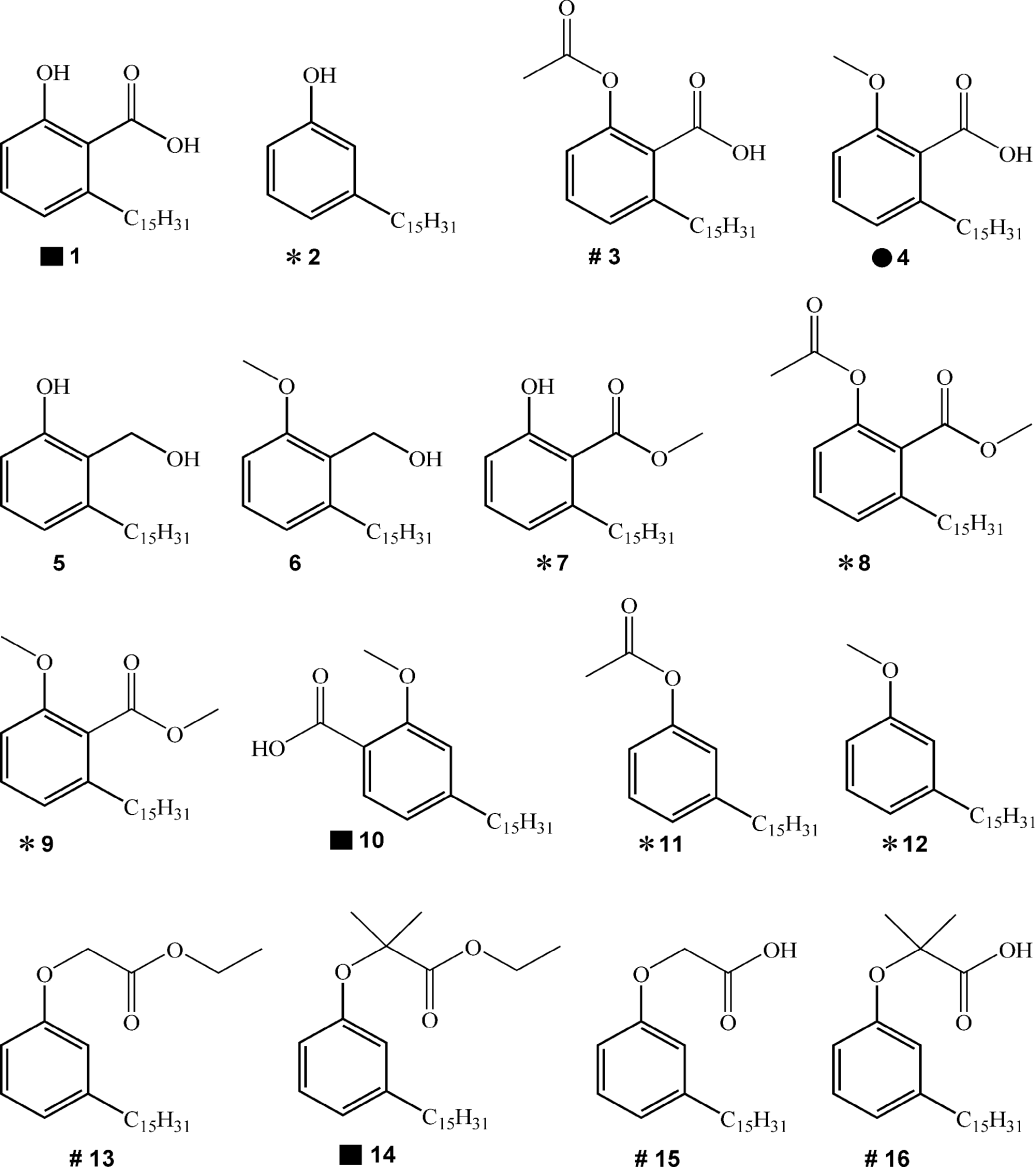
Chemical structures of anacardic acids and cardanols derivatives. * No effect on PPAR isoforms; #PPAR pan agonists; ■ dual (PPAR_α_ and PPAR_*y*_) agonist; ● PPAR_α_ selective agonist. PPAR activity for compounds 5 and 6 has not been reported.

To synthesize compounds **11** and **12**, **2** was converted to the acetyl and methoxy derivatives using similar procedures applied in the synthesis of compounds **9** and **3**. Compound **2** was also transformed into the α-phenoxyalkyl esters **13** and **14**. Finally, the ethyl esters **13** and **14** were hydrolyzed to the α-phenoxyalkyl acids **15** and **16** (Scheme 2).

**Scheme 2:**
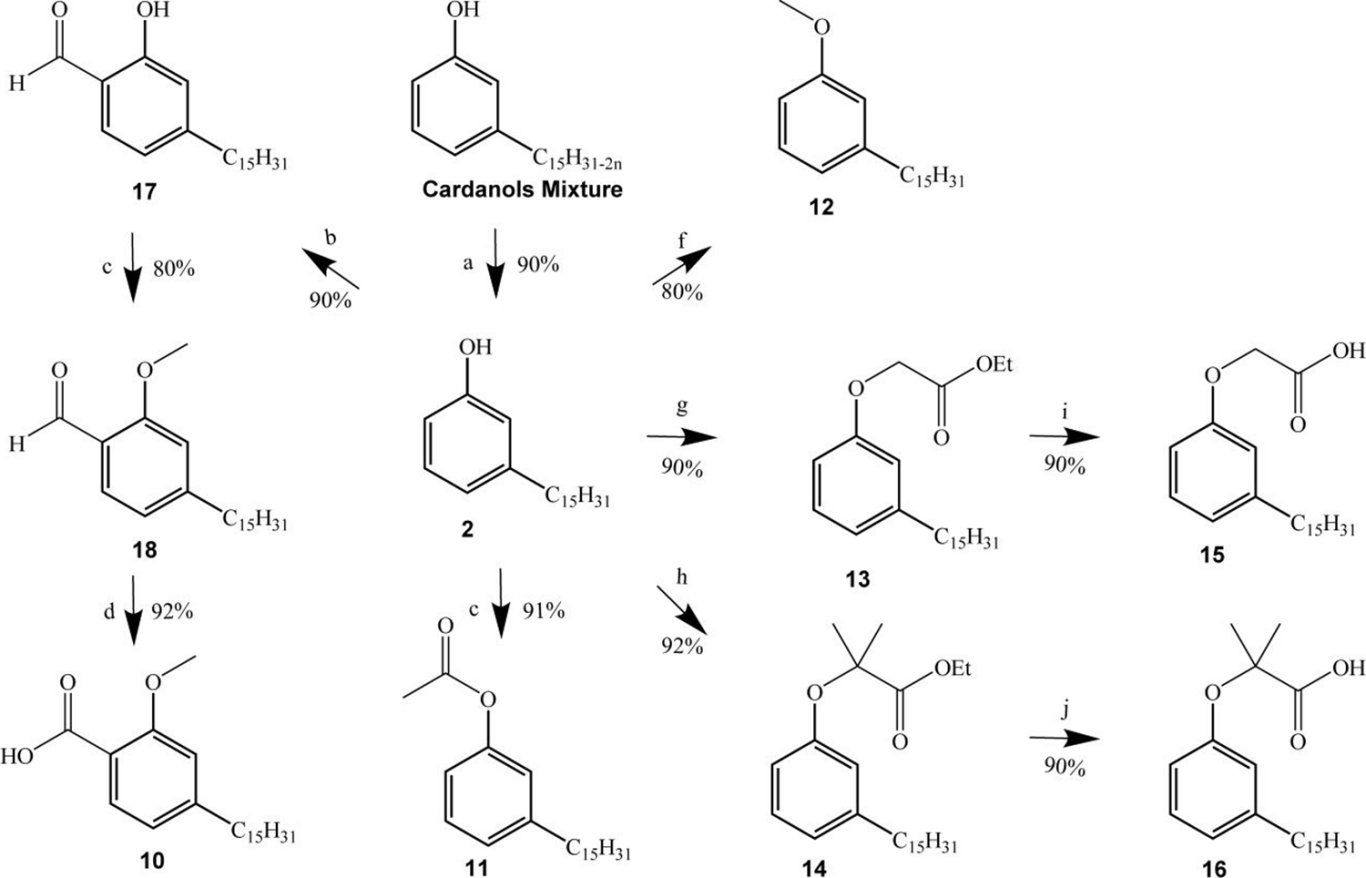
Synthesis of the Cardanols Derivatives^a^

a Reagents and conditions: a. H_2,_ Pd/C 10%, EtOH, r.t., 4 h; b. CH_2_O, MgBr_2,_ THF, reflux, 24; c. MeI, K_2_CO_3,_ Me_2_CO, 120 °C, 20 h; d. NaClO_2_ 1M, NaH_2_PO_4_ 1M, DMSO, CH_2_Cl_2,_ r.t., 16 h; e. AC_2_O, H_3_PO_4,_ MW (400 W), 3 min; f. MeI, K_2_CO_3_, Me_2_CO, 65 °C, 24 h; g. BrCH_2_CO_2_Et, K_2_CO_3_, Me_2_CO, r.t., 24 h; h. BrC­(CH_3_)_2_CO_2_Et, KI, K_2_CO_3_, MeCN, 82 °C, 24 h; i. LiOH, Aliquat, THF/H_2_O, r.t. Four h; j. LiOH, Aliquat, THF/H_2_O, 65 °C, 4 h.

#### 2-Methoxy-6-pentadecylphenylmethanol (**5**)

**9** (0.53 mmol) was dissolved in tetrahydrofuran (10 mL), followed by the addition of lithium aluminum hydride (2.12 mmol) in an ice bath. The mixture was heated at 66 °C for 18 h. The excess reducing agent was inactivated with methanol added dropwise, followed by the addition of 10% sodium hydroxide solution (1 mL) and distilled water (5 mL). Aluminum hydroxide was formed and neutralized with 10% aqueous hydrochloric acid to pH 4. The mixture was extracted with ethyl acetate (3 × 10 mL). The organic layer was washed with brine solution (10 mL) and dried with anhydrous sodium sulfate. The solvent was evaporated under reduced pressure, and the product was purified on silica gel (70–230 mesh) column chromatography, eluting with a gradient mixture of hexane and ethyl acetate (90:10 to 80:20), to afford compound 5 as a white solid. Yield 75%. mp 49–51 °C. IR (film, cm^–1^): 3385, 2919, 2847, 1590, 1466, 1437, 1320, 1268, 1091. ^1^H NMR (500 MHz, CDCl_3_) δ 7.20 (t, *J* = 7.8 Hz, 1H), 6.82 (d, *J* = 7.4 Hz, 1H), 6.77 (d, *J* = 7.95 Hz, 1H), 4.75 (s, 2H), 3.87 (s, 1H), 2.69 (t, *J* = 7.6 Hz, 2H), 1.57 (br, 2H), 1.27 (br, 29H), 0.89 (t, *J* = 6.85 Hz, 3H). ^13^C NMR (125 MHz, CDCl_3_) δ: 158.5, 142.8, 128.7, 127.1, 122.5, 108.3, 57.5, 55.6, 33.4, 32.3, 32.1, 29.9–29.6, 22.9, 14.3. HRMS (ESI-TOF): *m*/*z* [(M + Na)^+^] calculated for 371.2921, found 371.2926.

#### 2-Hydroxy-6-pentadecylphenylmethanol (**6**)

In a 100 mL flask were added 0.44 g of LiAlH_4_ (11.48 mmol) and THF (10 mL). In an ice bath with slight magnetic stirring, to the flask was added, dropwise, a solution of 1 (1.0 g, 2.87 mmol) in anhydrous THF (30 mL). After complete addition, the reaction was refluxed with heating in an oil bath at 110 °C with a condenser cooling system at 0 °C for 24 h. After this period, the excess of the reducing agent was inactivated by the dropwise addition of methanol in an ice bath. Then, 10% NaOH solution (2 mL) and distilled water (1 mL) were added, leading to the formation of aluminum hydroxide, and after 10 min, the reaction was acidified with 10% HCl solution until pH 1. The mixture was extracted with ethyl acetate (3 × 10 mL), and the combined organic phases were washed with brine (30 mL) and dried over anhydrous sodium sulfate. The solvent was removed under reduced pressure, and the product was purified on silica gel (70–230 mesh) column chromatography, eluting with a gradient mixture of hexane and ethyl acetate (90:10 to 40:60), giving compound **6** as a white solid. Yield 90%. mp 60–62 °C. IR (KBr, cm^–1^): 3526, 3190, 2919, 2852, 1466, 1364, 1260. ^1^H NMR (300 MHz, CDCl_3_) δ 7.48 (sl, 1H), 7.11 (t, *J* = 7.7 Hz, 1H), 6.72 (t, *J* = 8.3 Hz, 2H), 4.93 (s, 2H), 2.57 (t, *J* = 7.7 Hz, 2H), 1.63 (sl, 1H), 1.49 (m, 2H), 1.26 (m, 24H), 0.89 (t, *J* = 6.7 Hz, 3H); ^13^C NMR (75 MHz, CDCl_3_) δ 156.7, 141.2, 128.9, 122.6, 121.6, 114.5, 60.2, 33.3, 31.9, 31.8, 29.7–29.4, 22.7, 14.1.

### 2.2 Biological Assays

#### 2.2.1 Expression and Purification of PhzD from *P. aeruginosa* (*Pa*PhzD)

Cells of *E. coli* strain BL21­(DE3) containing the plasmid encoding for *Pa*PhzD from *P. aeruginosa* ^47^ were inoculated into 10 mL of sterile LB broth supplemented with kanamycin (50 μg/mL) and kept under constant agitation (180 rpm) at 37 °C for 16 h. The cell suspension was diluted (1:100) in sterile LB broth supplemented with kanamycin (50 μg/mL) and kept under constant shaking (180 rpm) at 37 °C until OD_600 nm_ = 0.6. At this point, the temperature was lowered to 18 °C, and IPTG (1 mM) was added to the culture, which was kept at constant shaking (180 rpm) for another 24 h.

The cells were recovered by centrifugation (16,000*g* at 4 °C for 30 min) and resuspended in Tris–HCl buffer pH 8.0 (50 mM) containing NaCl (100 mM), 1,4-dithiothreitol (1 mM), and imidazole (20 mM). Phenylmethanesulphonyl fluoride (1 mM) was added to the cell suspension immediately before mechanical lysis by sonication (15 cycles of 15 s at 10 W, with 30 s intervals), which was carried out in an ice-cold bath. The soluble fraction of the lysate was recovered by centrifugation (14.500*g* at 4 °C for 30 min), filtered, and loaded on a Ni-NTA column, previously equilibrated with 20 column volumes (CV) of buffer A [Tris-HCl buffer pH 8.0 (50 mM) containing NaCl (100 mM) and DTT (1 mM)]. The contaminants were eluted with 10 CV of buffer A, followed by an increasing gradient of imidazole (5 CV of 20–150 mM). Finally, *Pa*PhzD was eluted with buffer A supplemented with 250 mM imidazole. The fractions containing the protein were pooled together and then diluted (1:10) in Tris–HCl buffer, pH 8.0 (50 mM) containing NaCl (100 mM) and 1,4-dithiothreitol (1 mM). Following concentration by centrifugation, using an Amicon 30 kDa, at 4 °C and 3500 rpm, the dilution-concentration cycle was repeated three times. All purification steps were monitored with 12% polyacrylamide gel electrophoresis (SDS-PAGE), and the final protein concentration was evaluated by measuring the UV/vis absorbance at 280 nm (theoretical extinction coefficient of 1.635 M^–1^ cm^–1^ according to the ProtParam server, available at (http://web.expasy.org/protparam/). Imidazole-free *Pa*PhzD was stored at −80 °C in the presence of 30% glycerol.

#### 2.2.2 Thermal Shift Assays (TSA) Optimization

Thermal shift assays were performed on an Applied Biosystems 7500 RT-PCR (Applied Biosystems, Foster City, CA USA). All experiments were carried out in triplicate with 1 °C per minute increments in temperatures that range from 25 to 85 °C in 96-well PCR plates (PCR plates 96-well, BioRad), sealed with transparent capping strips (Flatcap strips, BioRad). The fluorescence of SYPRO Orange (Invitrogen S6650) was monitored (excitation wavelength, 492 nm; emission wavelength, 610 nm). Before the compounds’ screening, protein and DMSO concentrations were evaluated to improve *Pa*PhzD stability throughout the assay.

The protein concentration required for an optimal signal-to-noise ratio (>5-fold) was evaluated by analysis of the raw fluorescence curves at different *Pa*PhzD concentrations (1–5 μM). Then, the effect of DMSO (0–10%) over *Pa*PhzD *T*_m_ was evaluated at the optimal protein concentration. Fluorescence raw data were recorded using Applied Biosystems 7500 Software v2.0 and then processed with NAMI software^48^ to calculate *T*_m_ values by the first-derivative method.

#### 2.2.3 LDT Compounds Biological Evaluation by TSA

LDT compounds were screened at a final concentration of 50 μM. Briefly, 1 μL of each compound (1 mM DMSO stock solution) was added to a mixed solution containing 5 μM *Pa*PhzD diluted in 50 mM Tris–HCl buffer (pH 8.0) containing 100 mM NaCl, 1 mM DTT, and SYPRO Orange dye (Invitrogen S6650) (1:100 dilution). Each compound was assayed in triplicate, and Δ*T*_m_ values were calculated by comparison to reference wells that had 1 μL DMSO instead of the LDT compounds. Differences between *T*_m_ values (Δ*T*_m_) were considered statistically significant when *p* < 0.01, according to the one-way ANOVA followed by Dunnett’s post-test for multiple comparisons, available in GraphPad Prism 9.0 software (GraphPad Software, San Diego, CA, USA, www.graphpad.com).

Compounds with Δ*T*_m_ values statistically different from the reference were also assayed at different concentrations (ranging from 6.25 to 100 μM) to evaluate the concentration–response behavior. The Δ*T*_m_ versus concentration plots were generated using the nonlinear regression method available in GraphPad Prism version 9.0.

#### 2.2.4 LDT Compounds Biological Evaluation with *Pa*PhzD Covalently Bound to Fluorescein Isothiocyanate

*Pa*PhzD (2.1 mg/mL) was incubated with 6 mg/mL fluorescein isothiocyanate (FITC) (Sigma-Aldrich 46950) (1:1) for 2 h at 25 °C. Next, the reaction mixture was eluted on a Hi-Trap HP desalting column (GE Healthcare) with 2 CV of 50 mM HEPES buffer (pH 8.0). The collected fractions were quantified at two different wavelengths (280 and 495 nm), and the molar ratio (Abs495/Abs280) ranging from 0.3 to 1.0 was employed for *K*_d_ calculation experiments.

Subsequently, the fluorescence intensity of 0.32 μM of FITC-labeled *Pa*PhzD was measured on a real-time thermocycler (Applied Biosystems 7500) equipped with the filter FAN (excitation wavelength: 498 nm; emission wavelength: 530 nm) for 10 min in the presence of different concentrations of each LDT compound (ranging 0.7–100 μM), previously diluted in DMSO.

The fluorescence raw data were recorded using Applied Biosystems 7500 Software v2.0, then exported to GraphPad Prism version 9.0 software to build fluorescence intensity × concentration plots and calculate *K*_d_ values of the ligands by the nonlinear regression method (3-parameter fit). All experiments were carried out in triplicate using a 96-well PCR plate, manually sealed with transparent capping strips (Flatcap strips, BioRadVR).

#### 2.2.5 Screening of PYO Production Inhibitors

*P. aeruginosa* cells (ATCC 27853) were inoculated by depletion on King’s A agar, and the plates were incubated at 30 °C for 24 h. Isolated colonies were collected and resuspended in sterile saline solution (0.85%) until turbidity equivalent to optical density (OD) at 600 nm, equal to 0.3 (1.5 × 10^8^ CFU/mL). This bacterial suspension was diluted 10-fold and used to inoculate King’s A broth.

The potential inhibitors, previously solubilized in DMSO, were added to the culture medium after the inoculation (final concentration = 100 μM). The control group was cultured in the presence of an equivalent volume of DMSO but without the presence of potential inhibitors. The cell suspension was maintained at 30 °C under constant agitation (180 rpm) for 24 h in a 24-well plate. After this period, the OD_600_ was measured to evaluate bacterial growth, and in parallel, after being subjected to centrifugation, the supernatant was read in a 96-well plate at a wavelength of 691 nm. The sterile medium used for culturing the microorganism was used as a positive control, and culture medium containing 1% DMSO was used as a negative control.

#### 2.2.6 Biofilm Assay

Exponential phase cultures of *P. aeruginosa* PA14 were diluted to 5 × 10^5^ colony forming units (CFU) in 48-well plates with LB containing each compound (1, 3, and 5) at 100 μM final concentrations or 0.1% DMSO (v/v) as a negative control. The plate was incubated steadily at 30 °C for 20 h inside a plastic bag to avoid evaporation. After the incubation, the OD_600_ of each well was measured in a plate reader (SpectraMax Paradigm). The planktonic bacteria were removed by turning the plate over the waste, followed by three steps of washing with distilled water. After drying, a solution of 1% crystal violet was added to each well and incubated at room temperature for 30 min to stain the attached cells forming a ring at the air–liquid interface. The crystal violet solution was removed, and the plate was washed using the same procedure. The dye was solubilized with 30% acetic acid for 30 min at room temperature. The Abs_550_ was measured, and the value was divided by the OD_600_ for each well. A total of 24 replicates were made for each treatment (6 wells per plate, four plates)^49^

#### 2.2.7 Swarming Assay

Swarming assays were performed as described,^50^ using 6-well plates. Briefly, 3 μL of culture (OD_600_ = 3.0) was inoculated in the center of modified M9 (20 mM NH_4_Cl; 12 mM Na_2_HPO_4_; 22 mM KH_2_PO_4_; 8.6 mM NaCl; 1 mM MgSO_4_; 1 mM CaCl_2_ 2 H_2_O; 11 mM glucose; 0.5% casamino acids (Difco)) with 0.5% of Bacto-agar (Difco), containing the respective compounds (100 μM final concentration, diluted in DMSO). Plates were incubated inside plastic bags to avoid evaporation at 30 °C for 16 h. After this period, pictures were taken, and the area covered by the colonies was measured using ImageJ.^50^ All assays were performed with four replicates, and 0.1% DMSO (v/v) was employed as a negative control.

### 2.3 Statistical Analysis

#### 2.3.1 Thermal Shift Assays (TSA)

All data are expressed as means ± standard deviations, based on technical triplicates. Differences between *T*_m_ values (Δ*T*_m_) were considered statistically significant when *p* < 0.01, according to the one-way ANOVA followed by Dunnett’s post-test for multiple comparisons, available in GraphPad Prism 9.0 software (GraphPad Software, San Diego, CA, USA, www.graphpad.com).

#### 2.3.2 PYO Production, Biofilm, and Swarming Assay

All data are expressed as means ± standard deviations, based on technical triplicates for pyocyanin inhibition and swarming assays, or 12 technical replicates from two biological replicates for biofilm assays. For comparisons among three groups, statistical analyses were carried out using one-way ANOVA followed by Dunnett’s post-test. Differences were considered statistically significant at *p* < 0.05. All analyses were performed using GraphPad Prism version 9.0 (GraphPad Software, San Diego, CA, USA).

To account for the effect size in the phenotypic assays, the practical significance of the results was assessed by Cohen’s d (eq 1),^51^ which was computed as the standardized mean difference. This effect size was calculated by subtracting the control mean from the treatment mean and dividing by the pooled standard deviation derived from replicate values and sample sizes (eq 1).

$$d=\frac{{\overset{®}{x}}_{2}-{\overset{®}{x}}_{1}}{s_{P}}$$ 1

where *x*_2_ is the mean of replicates for each compound within each treated group, *x*_1_ represents the mean of replicates for the control group, and *s*_p_ is the pooled standard deviation calculated as shown in eq 2:

$$s_{p}=\sqrt{((n_{1}-1)S_{1}^{2}+(n_{2}-1)S_{2}^{2})/n_{1}+n_{2}-2}$$ 2

Here, *S*_1_ and *S*_2_ are the standard deviations of the control and treated groups, respectively, and *n*_1_ and *n*_2_ are the number of replicates for the control and treated groups, respectively.

## 3 Results and Discussion

The observation that the mixture of anacardic acids found in *Amphipterygium adstringens*, whose aromatic ring displays chemical similarities to the *Pa*PhzD substrate (2-amino-2-desoxyisochorismic acid—ADIC), decreases PYO production^52^ prompted us to explore an LDT series, which had previously undergone investigation of its peroxisome proliferator-activated receptor (PPAR) binding profile,^45^ to investigate whether this molecular scaffold would bind to *Pa*PhzD. As human PPAR and PhzD from *P. aeruginosa* share no significant sequence or structural similarity, our hypothesis was that dissimilar structure–activity relationships might emerge and a novel lead compound be identified. In order to achieve this goal, we selected six compounds that do not activate PPAR receptors (**2**, **7**, **8**, **9**, **11**, and **12**), four with pan-agonist activity on human PPAR isoforms (**3**, **13**, **15**, and **16**), three dual (PPARα and PPARγ) agonists (**1**, **10**, and **14**), and one selective PPARα agonist (**4**) to be screened as putative *Pa*PhzD inhibitors (Figure 2.

### 3.1 Binding of LDT to PhzD Using Thermal Shift Assays

Unfortunately, neither the substrate (2-amino-2-deoxyisochorismate—ADIC) nor the product ((5S,6S)-6-amino-5-hydroxy-1,3-cyclohexadieve-1-carboxylic acid—DHHA) of the reaction catalyzed by *Pa*PhzD is amenable to perform direct kinetic-assay measurements. We employed thermal shift assays (TSA) to perform the initial screening of the putative *Pa*PhzD ligands. The first step was to evaluate the effect of protein concentration and DMSO on the signal-to-noise ratio and *Pa*PhzD stability (Figure 1S). Our results show that at least 5 μM *Pa*PhzD is required to give a significant change in fluorescence. Thus, an eventual quenching of the signal by ligand binding would not compromise the quality of the results. DMSO 5.0% (v/v) did not significantly affect *Pa*PhzD *T*_m_ (Figure 1S).

First, we evaluated the impact of the pentadecyl salicylic derivatives (**1**, **3**, **4**, and **10**) at a single concentration (50 μM) on *Pa*PhzD *T*_m_ (Figure 3). All compounds with an unprotected carboxyl moiety resulted in a statistically significant shift (*p* < 0.05) in *Pa*PhzD *T*_m_ values (Δ*T*_m_ ranging from −4.7 °C (3) to −1.2 °C (10)) compared to the control. Esterification of the carboxyl groups produced compounds (**7**, **8**, and **9**) that did not shift *Pa*PhzD *T*_m_ (Δ*T*_m_ < 0.5 °C). The comparison of Δ*T*_m_ values between the regioisomers **4** (−3.3 °C) and **10** (−1.2 °C) also supports the importance of the carboxyl group in *Pa*PhzD binding. A similar biological profile was observed for 6-oxa isosteres of anacardic acids, which are low micromolar inhibitors (IC50 2–5 μM) of bacterial two-component regulatory systems (TCS), KinA/SPOOF from *Bacillus subtilis* and NRII/NRI from *E. coli*, when the carboxyl group is free, but lose potency (100X) upon esterification^53^ For those isosteres, it has also been claimed that an additional phenolic OH, vicinal to the COOH, is crucial for TCS inhibition, as compounds bearing only the acidic group are much less potent (IC50 > 88 μM).^53^ This observation does not apply to our data set as compounds **3**, **4**, and **10** have the hydroxyl protected.

**Figure 3:**
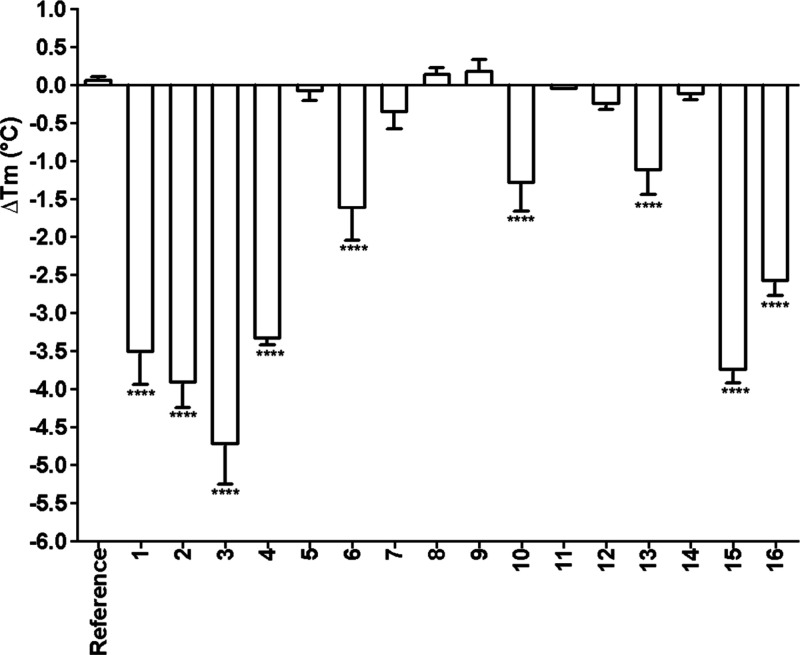
Single-concentration screening of LDT compounds (50 μM) against *Pa*PhzD (5 μM) using a thermal shift assay (TSA). Melting temperatures (*T*_m_) were determined with NAMI software using the first-derivative method, and Δ*T*_m_ values were calculated relative to reference wells containing DMSO. Each bar represents the mean Δ*T*_m_ value for LDT compounds from a single experiment performed in triplicate, and error bars indicate standard deviations. Statistical significance was evaluated by one-way ANOVA followed by Dunnett’s post-test (*p < 0.05, **p < 0.01, ***p < 0.001) (GraphPad Prism 9.0, GraphPad Software, San Diego, CA, USA).

In general, positive values of Δ*T*_m_ indicate that the ligand binds to the protein in its native conformation and stabilizes the native state. Contrastingly, negative values of Δ*T*_m_ suggest that the ligand binds preferentially to a less populated conformational state (e.g., a partially unfolded state or non-native state) of the protein.^45,54^ While the second profile is observed for compounds **3**, **4**, and **10**, they have the opposite effect on PPAR *T*_m_.^45^ For instance, compound **3** (LDT13) at 50 μM increases PPAR LBDs *T*_m_ values by up to +17 °C (higher than the *T*_m_ of DMSO control groups^45^). The negative shift seen in this work suggests that anacardic acid derivatives make protein unfolding easier, as described by Cimmperman et al.,^54^ or they bind to an allosteric site.

Another notable distinction between the binding profiles of the LDT compounds discussed here and the 6-oxa isosteres reported by Kanojia et al.^53^ is observed with cardanol derivative **2**. This compound is inactive (IC50 > 500 μM) against KinA/SPOOF from *B. subtilis* but causes a significant shift in *Pa*PhzD *T*_m_ (Δ*T*_m_ = −3.8 °C). Both acetylation (**11**) or methylation (**12**) of the phenol group result in compounds that do not alter *Pa*PhzD *T*_m_ values (Δ*T*_m_ < 0.5 °C). Conversely, alpha-phenoxyacid derivatives bearing a free carboxyl group distal from the aromatic ring (**15** Δ*T*_m_ = −3.8 °C and **16** Δ*T*_m_ = −2.5 °C) have a significant impact on *Pa*PhzD *T*_m_. Upon esterification to ethyl alpha-phenoxy esters, the effect on *Pa*PhzD *T*_m_ is either lost (**14** Δ*T*_m_ < 0.5 °C) or diminished (**13** Δ*T*_m_ = −1.1 °C). This outcome supports the significance of the carboxyl moiety in *Pa*PhzD binding, even if it is not directly on the aromatic ring. Results from an orthogonal assay, with covalently labeled *Pa*PhzD (discussed below), suggest the distal carboxyl group (COOH), by itself, is not enough to afford high-affinity ligands. Additionally, it is worth mentioning that compound **13** (LDT15) also induces a negative thermal shift in the LBDs of PPARα and PPARγ, which has been attributed to an interaction in an allosteric binding site.^55^

The TSA results also suggest a detrimental effect of the *gem*-dimethyl moiety on *Pa*PhzD affinity (**13**, Δ*T*_m_ = −1.2 °C vs **14**, Δ*T*_m_ = −0.1 °C; **16**, Δ*T*_m_ = −2.6 °C vs **15**, Δ*T*_m_ = −3.8 °C), likely due to steric hindrance that prevents the distal carboxyl group from interacting with *Pa*PhzD. Although **14** (LDT408) also displays no effect on the *T*_m_ values for all three PPARs,^55^ the fact that **13** affects them (see previous paragraph) suggests that the loss of binding is due to esterification rather than the additional bulkiness of the gem-dimethyl moiety.

Lastly, the necessity of the alkyl chain (C_15_H_31_ moiety) for binding was confirmed by assessing the effect of four benzoic acid derivatives with hydrogen in the equivalent position on the thermal stability of *Pa*PhzD (Δ*T*_m_ < 0.5 °C at 500 μM Figure 3S).

To exclude compounds with unspecific binding mechanisms, such as aggregators^56^ and PAINS,^57^ we investigated whether the effect of LDT compounds on *Pa*PzhD thermal stability is concentration-dependent. Among the nine compounds displaying a statistically significant negative shift in *Pa*PhzD *T*_m_ values (*p* < 0.05, Δ*T*_m_ ranging from −4.7 to −1.1 °C compared to the control, at 50 μM), 4 out of 6 compounds bearing a carboxyl moiety (**1**, **3**, **4**, **15**), and the compound with the phenolic OH (**2**), which is a much weaker acidic group, showed a clear concentration–response behavior within the tested concentrations (Figure 4). Considering the p*K*_a_ values of these groups (approximately 2–4 and 9–10, respectively) and the buffer used in the assay (pH = 8.0), it is reasonable to assume that the carboxyl group is almost completely ionized, which supports the requirement for a negatively charged group or a hydrogen bond acceptor for *Pa*PhzD binding. On the other hand, the phenolic OH group should be mainly unionized. Although it cannot be considered a negatively charged group, it is possible that it acts as a hydrogen bond donor or acceptor.

**Figure 4:**
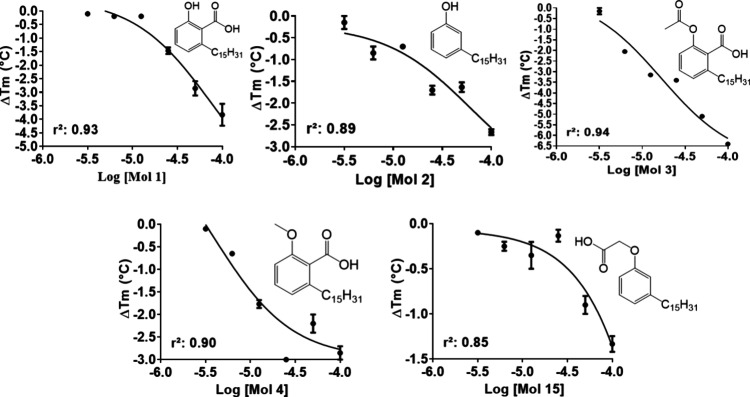
Concentration–response behavior of LDT compounds that decreased *Pa*PhzD thermal stability. The effect of each compound on the *Pa*PhzD melting temperature (*T*_m_) was evaluated across concentrations ranging from 6.25 to 100 μM. Δ*T*_m_ values were calculated relative to reference wells containing DMSO, and concentration–response curves were generated by plotting Δ*T*_m_ values against compound concentration and fitting the data using the nonlinear regression model available in GraphPad Prism 9.0 (GraphPad Software, San Diego, CA, USA).

Although other compounds might also exhibit a concentration–response behavior at higher concentrations, it could not be observed due to the solubility limits of the LDT compounds under the assay conditions (i.e., 5% DMSO concentration).

One might still argue that these five remaining compounds cannot be considered as *bona fide* hits, as Δ*T*_m_ values are not directly correlated to the compound’s affinity.^52^ Although Bai and co-workers^200^ have shown that the folded/unfolded protein ratio, at temperatures close to the protein *T*_m_, can be exploited to calculate *K*_d_ values, the algorithm provided by them is not compatible with compounds that reduce the protein *T*_m_ value (negative Δ*T*_m_). To overcome this limitation, an orthogonal binding assay using covalently modified *Pa*PhzD was carried out. Accordingly, 5′-fluorescein isothiocyanate-labeled *Pa*PhzD (*Pa*PhzD-FITC) had its fluorescence monitored at 25 °C, in the absence and presence of the initial hits, and compounds **2**, **3**, **4**, and **15** displayed the expected S-shaped curve (Figure 5).

**Figure 5:**
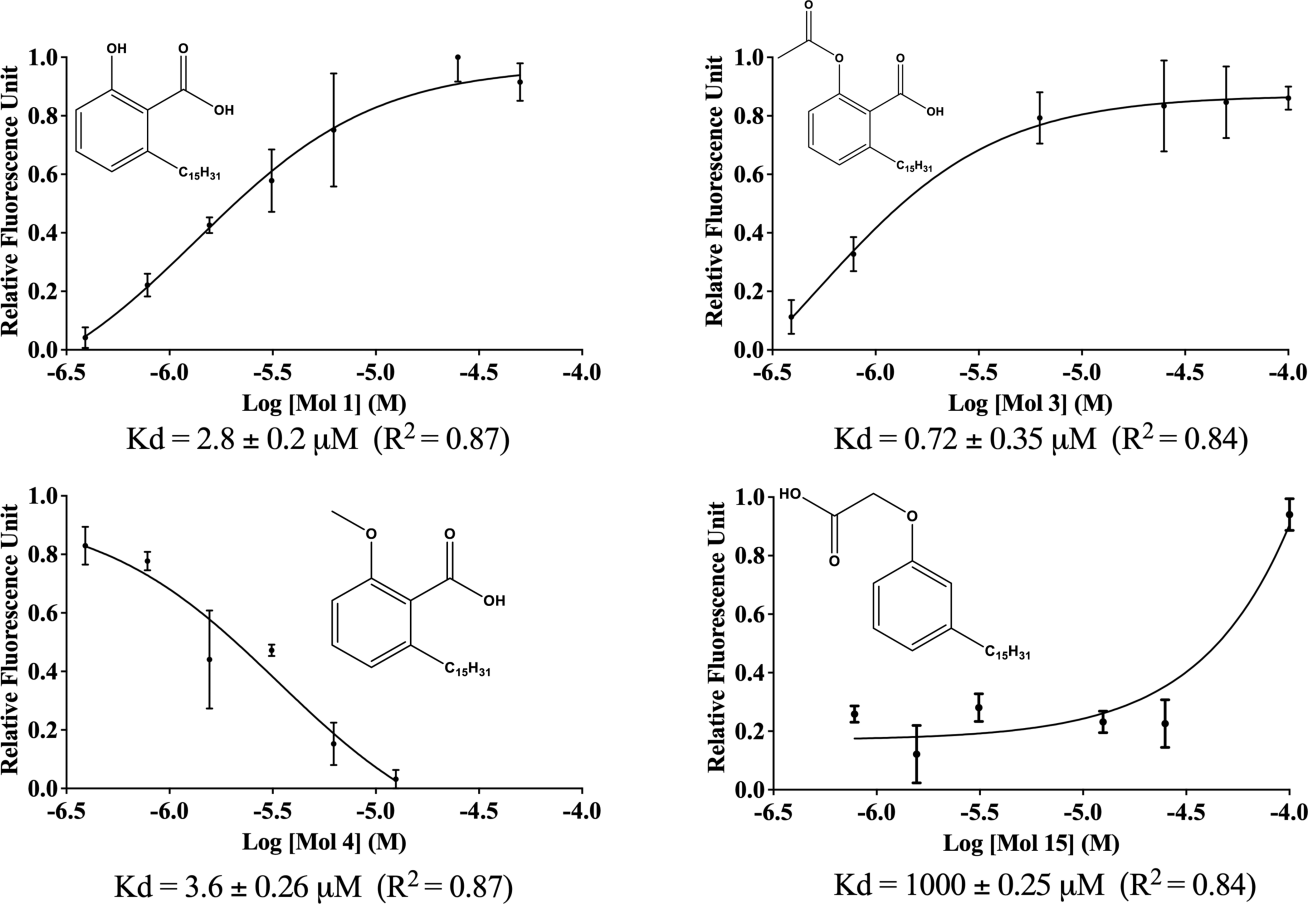
Fluorescence binding curves showing the effect of varying concentrations of compounds 2, 3, 4, and 15 on the fluorescence signal of covalently labeled *Pa*PhzD. Dissociation constants (*K*_d_) were determined using three-parameter nonlinear regression, as available in GraphPad Prism v9.0. Values represent the mean ± standard deviation of three technical replicate.

The comparison of *K*_d_ values for LDT **3**, **4**, and **15** (0.72, 3.6, and 1000 μM, respectively) suggests that the carboxylate of the alpha-phenoxyacids does not guarantee high-affinity binding to *Pa*PhzD.

On the other hand, for low to submicromolar affinity binding to *Pa*PhzD, a hydrogen bond acceptor directly attached to the ring is essential, as represented by the carboxylate group (3: *K*_d_ = 0.72 μM, 4: *K*_d_ = 3.6 μM) or the phenolic OH group (2: *K*_d_ = 2.8 μM).

Although docking studies might elucidate which *Pa*PhzD residues mediate these interactions and clarify structure–activity relationships distinct from those of LDT compounds binding to PPAR, their appropriate application demands a well-defined binding site (search space) where ligands are docked. While the negative shifts observed in TSA for LDT compounds are consistent with an allosteric binding profile, identifying the exact allosteric pockets of *Pa*PhzD is challenging, as different computational servers provide conflicting predictions regarding their locations (Figure 6S). Therefore, it is premature to employ docking simulations to validate the anacardic acid derivatives’ binding profile solely on the basis of the biological assay data reported here.

**Figure 6:**
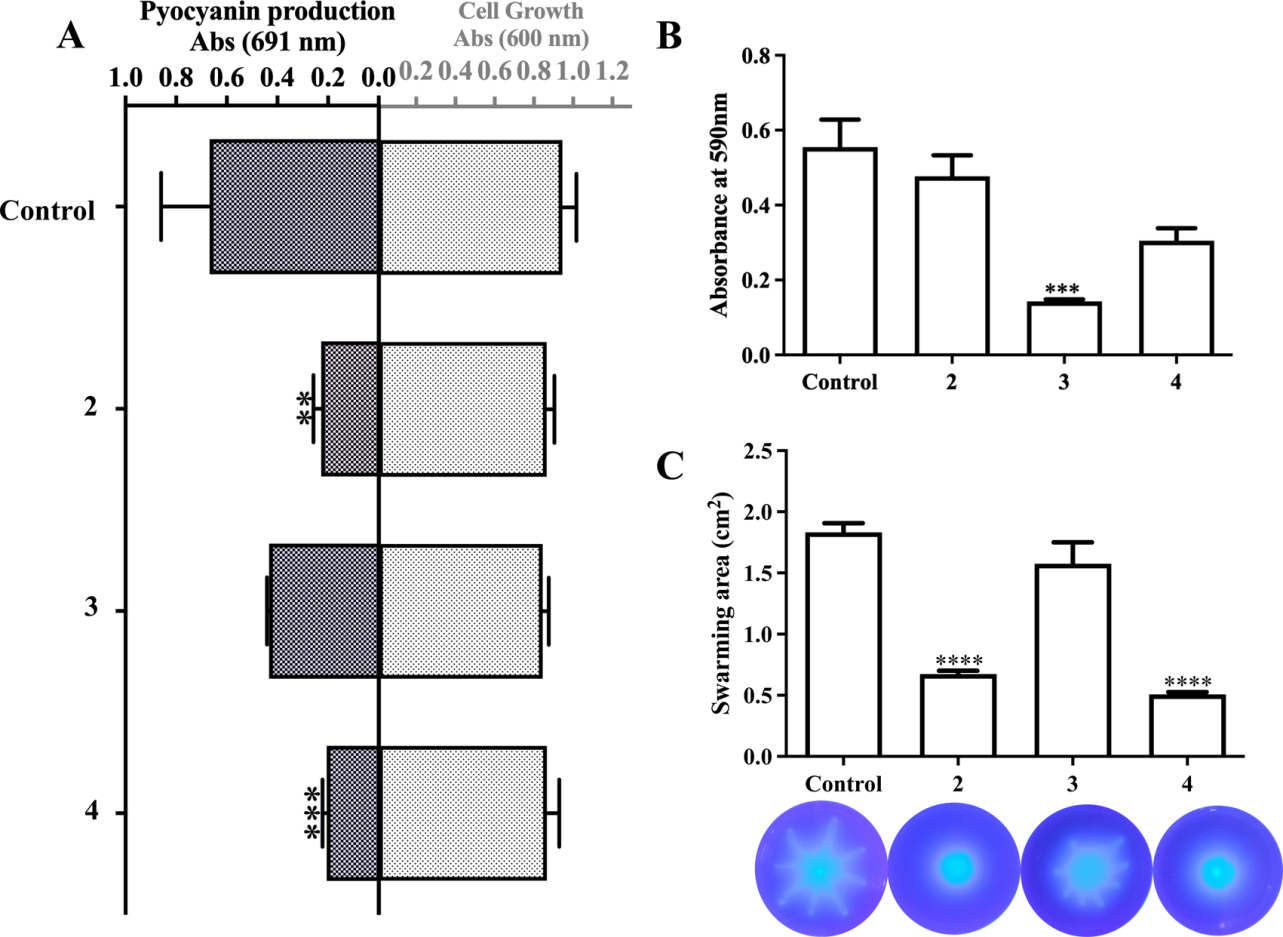
Effect of compounds **2**, **3**, and **4** (100 μM) on *P. aeruginosa*. (A) Growth and pyocyanin production. All experiments were carried out in triplicate, and DMSO (1% v/v) was employed as a negative control. The absolute absorption values were compared to a control culture (**p < 0.005, ***p < 0.001). (B) Biofilm initiation was evaluated in LB containing compounds **2**, **3**, or **4** by the crystal violet assay in 48-well plates. DMSO (0.1%) was added in the control assay. Error bars represent the standard deviation of three independent experiments (*n* = 3). ***p < 0.0001. (C) Swarming motility in the presence of LDT compounds. The compounds were added to the medium at a concentration of 100 μM, the cells were inoculated, and the swarming area was measured using ImageJ after 2 days and compared to a control with DMSO. Error bars represent the standard deviation of three independent experiments (*n* = 3). ****p < 0.0001. Statistical significance was evaluated by one-way ANOVA followed by Dunnett’s post-test (*p < 0.05, **p < 0.01, ***p < 0.001) (GraphPad Prism 9.0, GraphPad Software, San Diego, CA, USA).

Nevertheless, it is noteworthy that the fourth most probable allosteric pocket predicted by the Passer 2.0 server^58^ is situated near an allosteric site-forming residue (LEU120), as predicted by the StingAllo server,^59^ and aligns with consensus clusters (CS1 and CS7) identified by the FTMap server^60^ when the active site is masked. Since FTMap has been successfully employed to predict allosteric and cryptic binding pockets,^61,62^ these findings support the presence of a solvent-accessible pocket (Total SAS 151.55 Å^2^) (Figure 6S) as a plausible allosteric binding site for docking LDT compounds. While this pocket contains two basic residues (ARG41 and ARG105) that may interact with the carboxylate or phenolic OH group, experimental validation is essential to confirm whether this is indeed the authentic binding pocket for LDT compounds.

### 3.2 Phenotypic Assays

Although the biological activity of the mixture of anacardic acids over PYO has already been reported,^52^ up to this point, there was no evidence that compounds **2** (*p* = 0.0013, Cohen′d = −2.56), **3** (*p* = 0.0808, Cohen′d = −1.37), or **4** (*p* = 0.0009, Cohen′d = −2.69) would, in fact, decrease PYO production, since their binding to *Pa*PhzD might be overridden by cellular compensatory mechanisms, such as positive regulation of the operon that codes for *Pa*PhzD and related enzymes^45,54^ . Moreover, the SAR profile of LDT compounds that bind *Pa*PhzD, discussed above, is different from the ones described for 6-oxa isosteres of anacardic acids;^53^ consequently, their cellular activity cannot be easily predicted. To shed light on this matter, the effect of the most promising compounds (**2**, **3**, and **4**) on *P. aeruginosa* viability and PYO production was evaluated (Figure 6A). Compounds **2** and **4** decreased pyocyanin production with no significant effect on bacterial growth, whereas compound **3** had no effect on pyocyanin production when compared to that of the control. The lack of antimicrobial activity against a Gram-negative bacterium (*P. aeruginosa*) is in good agreement with that reported by Castillo-Juarez and co-workers for the mixture of anacardic acids^52^ and by Kubo and co-workers for anacardic acid derivatives with alkyl chains with 5–17 carbons.^63^

Considering that compound **3** has a 50× higher affinity than **4** for *Pa*PhzD, the lack of PYO production inhibition is likely due to pharmacokinetic issues.

To further investigate this matter, we predicted the pharmacokinetic properties of compounds **2**, **3**, and **4** using the ADMETlab 3.0 server.^64^ Both compounds **3** and **4** exhibit high predicted human intestinal absorption (HIA: 98.7 and 98.9%, respectively) and good permeability, with Caco-2 permeability values of 0.66 and 0.75. However, poor permeability across bacterial membranes has been implicated in the differing antimicrobial activity of anacardic acid derivatives against Gram-positive and Gram-negative bacteria.^59^ Moreover, *P. aeruginosa* expresses an outer membrane esterase selective for long-chain thioesters (C12–C18)^65^ that may hydrolyze the ester bond in compound **3**, converting it to compound **1**. Although compound **1** initially showed promising activity in thermal shift assays (TSA), it was ultimately discarded after orthogonal binding assays performed at a single temperature. Other drawbacks that must be addressed include the predicted high affinity to plasma proteins (88% plasma protein binding for compound **3** and 85% for compound **4**), as well as moderate to high elimination (plasma clearance: 4.43 mL/min/kg for compound **3** and 4.56 mL/min/kg for compound **4**).

Toxicity predictors and MTT viability assays (at 25 μM) support minimal cytotoxicity, with cell viability near 100% in H9c2 and HEK293 cells (Figure 8S Supporting Information).

The overall production of PYO is regulated by several quorum-sensing (QS) mechanisms,^66^ so one might argue that the previous results do not prove that *Pa*PhzD is the cellular target of compounds **2** and **4**. To further investigate the mechanism of action of those compounds, we assessed their effect on *P. aeruginosa* biofilm development, and motility was assessed.

Microbial biofilms increase the costs of drinking water treatment and can be found on medical and dental devices, as well as artificial organs, limiting their impact on human health, as bacteria in biofilms are more resistant to antibiotics and immune responses.^18,67^ Jagani et al.^68^ showed that both the mixture of anacardic acids and cardanols, at 4 μg mL^–1^, decrease biofilm formation, referred to as biofouling, by 50.5 and 52.9% respectively. The evaluation of compounds **2**, **3**, and **4** antibiofilm activity, assessed by a similar crystal violet assay (Figure 6B), shows that biofilm growth in the presence of either compound **2** or **4** is not significantly different the control, but compound **3** at 100 μM reduced biofilm mass to ∼ 30% of the control (*p* < 0.0001, Cohen’d = −2.29). In this biofilm initiation assay, the role of PYO as an electron acceptor is not relevant, as the cell biomass is at the medium–water interface and access to oxygen is not limited. Therefore, inhibition of *Pa*PhzD by compounds **2** and **4** is not expected to be directly limiting in biofilm initiation, unless QS system regulation is impacted by lower levels of PYO. Hence, the compound **3** inhibitory effect on biofilm production is unrelated to *Pa*PhzD.

PYO production is important for maintaining the redox balance in cells growing in oxygen-deprived conditions,^69^ and colonies deficient in PYO production may become wrinkled, increasing the surface area in contact with air. No effects on colony morphology were observed when bacteria were grown in medium containing compounds **2**, **3**, and **4**, discarding their role in disturbing the redox state under the conditions tested (not shown).

The swarming motility is a crucial feature in the early steps of biofilm formation and/or biofilm maturation.^70,71^ In fact, *P. aeruginosa* mutants with altered swarming motility display impaired biofilm formation,^70,72^ whereas swarming strains of *P. aeruginosa* have higher resistance to antibiotics than swimming cells.^73^ The surfactants secreted by *P. aeruginosa* cells generate a flow used to propel them, and if the surface tension is broken, motility does not occur properly due to the physical properties of the medium.^74^ Considering that *P. aeruginosa* swarming is strongly correlated with rhamnolipid production,^75,76^ Castillo-Juarez and co-workers reported a concentration-dependent decrease in this surfactant production when *P. aeruginosa* PA14 is exposed to the anacardic acid mixture.^52^ Since motility and biofilm assays had already been optimized for this strain, it was selected to evaluate the effect of compounds **2**, **3**, and **4** (Figure 6C). Swarming motility in the presence of compounds **2** (*p* < 0.0001, Cohen’d = −0.91) and **4** (*p* < 0.0001, Cohen’d = −0.95) was reduced to 65%, as compared to control conditions. Treatment with compound **3** (*p* = 0.2571, Cohen′d = 0.51) did not affect the coverage area but had a visible impact on dendrite formation (Supporting Information). Given the collective nature of swarming and the key role of QS in regulating this behavior, the compound 3 effect is compatible QS modulation. Therefore, swarming may be affected by LDT compounds because of their effect on the surface of the medium such as changes in hydrophilic/hydrophobic properties.

It is hard to separate the effect of each compound on the complex regulatory network of the bacteria. The interaction of compounds **2** and **4** with *Pa*PhzD is the probable cause of PYO reduction. Considering the role of PYO as a signaling molecule, this initial reduction could have downstream effects that lead to altered biofilm and swarming phenotypes.^77^ Other studies that identified compounds that reduce both swarming and PYO tracked this effect on interferences with RlhR and LasR.^78,79^ It is surprising to see an impact on swarming but not on biofilm, but it is not unprecedented, since other compounds have this effect.^80,81^ It is also possible that the impact is greater in the later stages of biofilm maturation, which is out of the range of the conditions used here. Compound **3** has no impact on PYO but reduces biofilm formation and causes misshaped swarming. As previously stated, it is possible that the compound’s low permeability might prevent it from entering the cell. Nevertheless, the presence of the molecule, even on the outside, can prevent cell attachment to the surface, leading to impaired biofilm formation.^82^ The same can be argued about swarming, which can be affected by additives that change the surface tension.^83,84^ Ultimately, the difference between the compound’s effects on phenotypes can be explained by **2** and **4** being able to enter the cell and interact with *Pa*PhzD, whereas compound **3** has an extracellular phenotypic action that is unrelated to this target.

This study used the well-characterized laboratory strain *P. aeruginosa* PA14 to balance practical constraints with biological relevance. Although PA14 is a lab strain, it retains high virulence and is frequently employed in comparative studies. Conversely, clinical isolates from chronic infections often exhibit reduced virulence, which could confound results (Winstanley et al 2016;^85^ Bhagirath et al., 2016 ^86^). While using a single strain imposes limitations, the phenotypes studied reflect conserved virulence traits regulated by core genomic pathways (Poulsen et al., 2019 ^87^). Therefore, our findings are likely applicable to acute infections where quorum sensing is essential, but caution should be exercised when extrapolating to chronic infections, given PA14’s weaker biofilm-forming capacity.

## 4 Conclusions

Thorough characterization of LDT compounds using two distinct in vitro assays led to the identification of the first micromolar ligands of *Pa*PhzD. This finding aligns with our hypothesis regarding distinct structure–activity relationship (SAR) requirements compared to those of LDT compounds acting as PPAR ligands. Further exploration of the biological profile revealed that compounds **2** and **4** possess favorable pharmacokinetics to cross the cell envelope and reduce both PYO production and biofilm development under the tested conditions. Despite exhibiting the highest affinity to *Pa*PhzD, compound **3** exclusively affected biofilm formation, indicating a need for fine-tuning its physicochemical properties.

## Supplementary Material



## Notes

Optimization of the thermofluor assay; gene expression, computational, cytotoxicity studies, and ^1^H and ^13^C NMR for compounds (PDF)

## #

Laboratório de Cristalografia de Proteínas, Center for the Research and Advancement of Fragments and Molecular Targets, Faculdade de Ciências Farmacêuticas de Ribeirão Preto, Universidade de São Paulo, Ribeirão Preto, SP, 14040-903, Brazil

## ∇

Laboratory of Host-Parasite Interaction and Epidemiology Gonçalo Moniz Institute, Fiocruz, Salvador, BA, Brazil.

## ○

McGill University, 845 Sherbrooke St. W., Montreal, QC, H3A 0G4, Canada.

## ◆

Novo Nordisk Foundation Center for Basic Metabolic Research, University of Brasilia.

## ¶

Medlife Produtos Laboratoriais, Universidade Católica de Brasília, Universidade de Brasília.

## Abbreviations

ADIC: 2-amino-2-deoxyisochorismate
AMR: antimicrobial resistance
ANOVA: analysis of variance
ATCC: American Type Culture Collection
CFU: colony forming units
CNSL: cashew nut shell liquid
DHHA: 6-amino-5-hydroxy-1,3-cyclohexadiene-1-carboxylic acid
DMSO: dimethyl sulfoxide
DTT: dithiothreitol
ESI: electrospray ionization
ESI-TOF: electrospray ionization time-of-flight
FITC: fluorescein isothiocyanate
HEPES: 4-(2-hydroxyethyl)-1-piperazineethanesulfonic acid
HRMS: high-resolution mass spectrometry
IL: interleukin
IPTG: isopropyl β-D-1-thiogalactopyranoside
LB: Luria–Bertani medium
NAMI: NAMI software
NMR: nuclear magnetic resonance
OD: optical density
PAINS: pan-assay interference compounds
PCR: polymerase chain reaction
PYO: pyocyanin
QS: quorum sensing
SAR: structure–activity relationship
SDS: sodium dodecyl sulfate
SDS–PAGE: SDS polyacrylamide gel electrophoresis
TEA: triethylamine
THF: tetrahydrofuran
TLC: thin-layer chromatography
TSA: thermal shift assay
*T*_m_: melting temperature

## References

[1] European Centre for Disease Prevention and Control. Antimicrobial Resistance in the EU/EEA (EARS-Net) EU Targets on Antimicrobial Resistance; 2024. https://atlas.ecdc.europa.eu/.

[2] Singh S. B., Young K., Silver L. L. (2017). What Is
an “Ideal”
Antibiotic? Discovery Challenges and Path Forward. Biochem. Pharmacol..

[3] Thorpe K. E., Joski P., Johnston K. J. (2018). Antibiotic-Resistant
Infection Treatment
Costs Have Doubled since 2002, Now Exceeding $2 Billion Annually. Health Aff.

[4] Luepke K. H., Suda K. J., Boucher H., Russo R. L., Bonney M. W., Hunt T. D., Mohr J. F. (2017). Past, Present, and
Future of Antibacterial
Economics: Increasing Bacterial Resistance, Limited Antibiotic Pipeline,
and Societal Implications. Pharmacotherapy.

[5] Tacconelli E., Carrara E., Savoldi A., Harbarth S., Mendelson M., Monnet D. L., Pulcini C., Kahlmeter G., Kluytmans J., Carmeli Y., Ouellette M., Outterson K., Patel J., Cavaleri M., Cox E. M., Houchens C. R., Grayson M. L., Hansen P., Singh N., Theuretzbacher U., Magrini N., Aboderin A. O., Al-Abri S. S., Awang Jalil N., Benzonana N., Bhattacharya S., Brink A. J., Burkert F. R., Cars O., Cornaglia G., Dyar O. J., Friedrich A. W., Gales A. C., Gandra S., Giske C. G., Goff D. A., Goossens H., Gottlieb T., Guzman Blanco M., Hryniewicz W., Kattula D., Jinks T., Kanj S. S., Kerr L., Kieny M. P., Kim Y. S., Kozlov R. S., Labarca J., Laxminarayan R., Leder K., Leibovici L., Levy-Hara G., Littman J., Malhotra-Kumar S., Manchanda V., Moja L., Ndoye B., Pan A., Paterson D. L., Paul M., Qiu H., Ramon-Pardo P., Rodríguez-Baño J., Sanguinetti M., Sengupta S., Sharland M., Si-Mehand M., Silver L. L., Song W., Steinbakk M., Thomsen J., Thwaites G. E., van der Meer J. W., Van Kinh N., Vega S., Villegas M. V., Wechsler-Fördös A., Wertheim H. F. L., Wesangula E., Woodford N., Yilmaz F. O., Zorzet A. (2018). Discovery, Research, and Development of New Antibiotics:
The WHO Priority List of Antibiotic-Resistant Bacteria and Tuberculosis. Lancet Infect Dis.

[6] Cooper M. A., Shlaes D. (2011). Fix the Antibiotics
Pipeline. Nature.

[7] Zhao P., Xue Y., Li X., Li J., Zhao Z., Quan C., Gao W., Zu X., Bai X., Feng S. (2019). Fungi-Derived Lipopeptide
Antibiotics Developed since 2000. Peptides.

[8] Novak R. (2011). Are Pleuromutilin
Antibiotics Finally Fit for Human Use?. Ann.
N.Y. Acad. Sci..

[9] Pechere J. (1996). Streptogramins. Drugs.

[10] Davies, J. Origins and Evolution of Antibiotic Resistance. Microbiología (Madrid, Spain). American Society for Microbiology (ASM), 1996; pp 9–16. 9019139

[11] Bountra, C. ; Lee, W. H. ; Lezaun, J. A New Pharmaceutical Commons: Transforming Drug Discovery. Oxford Martin Policy Paper 2017.

[12] Ali J., Rafiq Q. A., Ratcliffe E. (2018). Antimicrobial Resistance Mechanisms
and Potential Synthetic Treatments. Future Sci.
OA.

[13] Lyczak J. B., Cannon C. L., Pier G. B. (2000). Establishment of Pseudomonas Aeruginosa
Infection: Lessons from a Versatile Opportunist. Microbes Infect.

[14] Wagner V. E., Iglewski B. H. P. (2008). Aeruginosa Biofilms
in CF Infection. Clin. Rev. Allergy Immunol..

[15] Weinstein R. A., Gaynes R., Edwards J. R. (2005). Overview
of Nosocomial Infections
Caused by Gram-Negative Bacilli. Clinical Infectious
Diseases.

[16] Breidenstein E. B. M., de la Fuente-Núñez C., Hancock R. E. W. (2011). Pseudomonas Aeruginosa:
All Roads Lead to Resistance. Trends Microbiol.

[17] Bajire S. K., Shastry R. P. (2024). Synergistic Effects of COVID-19 and Pseudomonas Aeruginosa
in Chronic Obstructive Pulmonary Disease: A Polymicrobial Perspective. Mol. Cell. Biochem..

[18] Taylor P. K., Yeung A. T. Y., Hancock R. E. W. (2014). Antibiotic
Resistance in Pseudomonas
Aeruginosa Biofilms: Towards the Development of Novel Anti-Biofilm
Therapies. J. Biotechnol..

[19] Theuretzbacher U. (2017). Global Antimicrobial
Resistance in Gram-Negative Pathogens and Clinical Need. Curr. Opin. Microbiol..

[20] Nesher L., Rolston K. V. I., Shah D. P., Tarrand J. T., Mulanovich V., Ariza-Heredia E. J., Chemaly R. F. (2015). Fecal Colonization and Infection
with Pseudomonas Aeruginosa in Recipients of Allogeneic Hematopoietic
Stem Cell Transplantation. Transplant Infectious
Disease.

[21] Ahmed S. A. K. S., Rudden M., Smyth T. J., Dooley J. S. G., Marchant R., Banat I. M. (2019). Natural Quorum Sensing Inhibitors Effectively Downregulate
Gene Expression of Pseudomonas Aeruginosa Virulence Factors. Appl. Microbiol. Biotechnol..

[22] DIckey S. W., Cheung G. Y. C., Otto M. (2017). Different
Drugs for Bad Bugs: Antivirulence
Strategies in the Age of Antibiotic Resistance. Nat. Rev. Drug Discov.

[23] Mortzfeld F. B., Pietruszka J., Baxendale I. R. (2019). A Simple and Efficient Flow Preparation
of Pyocyanin a Virulence Factor of Pseudomonas Aeruginosa. Eur. J. Org. Chem..

[24] McGhee J. R., Denning G. M., Wollenweber L. A., Railsback M. A., Cox C. D., Stoll L. L., Britigan B. E. (1998). Pseudomonas
Pyocyanin
Increases Interleukin-8 Expression by Human Airway Epithelial Cells. Infect. Immun..

[25] Luo J., Dong B., Wang K., Cai S., Liu T., Cheng X., Lei D., Chen Y., Li Y., Kong J., Chen Y., Seleem M. N. (2017). Baicalin Inhibits
Biofilm Formation, Attenuates the Quorum Sensing-Controlled Virulence
and Enhances Pseudomonas Aeruginosa Clearance in a Mouse Peritoneal
Implant Infection Model. PLOS One.

[26] Al-Yousef H. M., Ahmed A. F., Al-Shabib N. A., Laeeq S., Khan R. A., Rehman M. T., Alsalme A., Al-Ajmi M. F., Khan M. S., Husain F. M. (2017). Onion Peel Ethylacetate
Fraction and Its Derived Constituent
Quercetin 40-O-β-D Glucopyranoside Attenuates Quorum Sensing
Regulated Virulence and Biofilm Formation. Front.
Microbiol..

[27] Heidari A., Noshiranzadeh N., Haghi F., Bikas R. (2017). Inhibition of Quorum
Sensing Related Virulence Factors of Pseudomonas Aeruginosa by Pyridoxal
Lactohydrazone. Microb Pathog.

[28] Chatterjee M., D’Morris S., Paul V., Warrier S., Vasudevan A. K., Vanuopadath M., Nair S. S., Paul-Prasanth B., Mohan C. G., Biswas R. (2017). Mechanistic Understanding of Phenyllactic
Acid Mediated Inhibition of Quorum Sensing and Biofilm Development
in Pseudomonas Aeruginosa. Appl. Microbiol.
Biotechnol..

[29] Wang Y., Zhang W. Z., Song L. F., Zou J. J., Su Z., Wu W. H. (2008). Transcriptome Analyses Show Changes in Gene Expression
to Accompany
Pollen Germination and Tube Growth in Arabidopsis1­[W]­[OA]. Plant Physiol..

[30] Saleem, M. Natural Products as Antimicrobial Agents - an Update. In Novel Antimicrobial Agents and Strategies; Wiley-VCH Verlag GmbH & Co. KGaA: Weinheim, Germany, 2014; pp 219–294.

[31] Zhao W., Cross A. R., Crowe-McAuliffe C., Weigert-Munoz A., Csatary E. E., Solinski A. E., Krysiak J., Goldberg J. B., Wilson D. N., Medina E., Wuest W. M., Sieber S. A. (2019). The Natural
Product Elegaphenone Potentiates Antibiotic Effects against Pseudomonas
Aeruginosa. Angewandte Chemie - International
Edition.

[32] Chong Y. M., How K. Y., Yin W. F., Chan K. G. (2018). The Effects of Chinese
Herbal Medicines on the Quorum Sensing-Regulated Virulence in Pseudomonas
Aeruginosa PAO1. Molecules.

[33] Maisuria V. B., Los Santos Y. L. De, Tufenkji N., Déziel E. (2016). Cranberry-Derived
Proanthocyanidins Impair Virulence and Inhibit Quorum Sensing of Pseudomonas
Aeruginosa. Sci. Rep..

[34] Harjai K., Kumar R., Singh S. (2010). Garlic Blocks
Quorum Sensing and
Attenuates the Virulence of Pseudomonas Aeruginosa. FEMS Immunol Med. Microbiol.

[35] Froes T. Q., Chaves B. T., Mendes M. S., Ximenes R. M., da Silva I. M., da Silva P. B. G., de
Albuquerque J. F.
C., Castilho M. S. (2021). Synthesis
and Biological Evaluation of Thiazolidinedione Derivatives with High
Ligand Efficiency to P. Aeruginosa PhzS. J.
Enzyme Inhib Med. Chem..

[36] Froes T. Q., Nicastro G. G., de Oliveira
Pereira T., de Oliveira Carneiro K., Alves Reis I. M., Conceição R. S., Branco A., Ifa D. R., Baldini R. L., Castilho M. S. (2020). Calycopterin, a
Major Flavonoid from Marcetia Latifolia, Modulates Virulence-Related
Traits in Pseudomonas Aeruginosa. Microb. Pathog..

[37] Froes T. Q., Baldini R. L., Vajda S., Castilho M. S. (2019). Structure-Based
Druggability Assessment of Anti-Virulence Targets from Pseudomonas
Aeruginosa. Curr. Protein Pept Sci..

[38] Froes T. Q., Guido R. V. C., Metwally K., Castilho M. S. (2020). A Novel Scaffold
to Fight Pseudomonas Aeruginosa Pyocyanin Production: Early Steps
to Novel Antivirulence Drugs. Future Med. Chem..

[39] Soares
Romeiro L. A., da Costa Nunes J. L., de Oliveira Miranda C., Simões Heyn Roth Cardoso G., de Oliveira A. S., Gandini A., Kobrlova T., Soukup O., Rossi M., Senger J., Jung M., Gervasoni S., Vistoli G., Petralla S., Massenzio F., Monti B., Bolognesi M. L. (2019). Novel Sustainable-by-Design HDAC
Inhibitors for the Treatment of Alzheimer’s Disease. ACS Med. Chem. Lett..

[40] de
Souza M. Q., Teotônio I. M.
S. N., de Almeida F. C., Heyn G. S., Alves P. S., Romeiro L. A. S., Pratesi R., de Medeiros Nóbrega Y. K., Pratesi C. B. (2018). Molecular Evaluation
of Anti-Inflammatory Activity of Phenolic Lipid Extracted from Cashew
Nut Shell Liquid (CNSL). BMC Complement Altern.
Med..

[41] Gomes
Júnior A. L., Islam M. T., Nicolau L. A. D., De
Souza L. K. M., Araújo T. de S.
L., Lopes De
Oliveira G. A., De Melo Nogueira K., Da Silva Lopes L., Medeiros J. V. R., Mubarak M. S., Melo-Cavalcante A. A. de C. (2020). Anti-Inflammatory,
Antinociceptive, and Antioxidant of Anacardic Acid in Experimental
Models. ACS Omega.

[42] M
Ashraf S., Rathinasamy K. (2018). Antibacterial and Anticancer Activity
of the Purified Cashew Nut Shell Liquid: Implications in Cancer Chemotherapy
and Wound Healing. Nat. Prod Res..

[43] Morais S. M., Silva K. A., Araujo H., Vieira I. G. P., Alves D. R., Fontenelle R. O. S., Silva A. M. S. (2017). Anacardic Acid Constituents from
Cashew Nut Shell Liquid: NMR Characterization and the Effect of Unsaturation
on Its Biological Activities. Pharmaceuticals
(Basel).

[44] Lomonaco D., Pinheiro Santiago G. M., Ferreira Y. S., Campos Arriaga Â.
M., Mazzetto S. E., Mele G., Vasapollo G. (2009). Study of Technical
CNSL and Its Main Components as New Green Larvicides. Green Chem..

[45] Sahin C., Magomedova L., Ferreira T. A. M., Liu J., Tiefenbach J., Alves P. S., Queiroz F. J. G., Oliveira A. S. D., Bhattacharyya M., Grouleff J., Nogueira P. C. N., Silveira E. R., Moreira D. C., Leite J. R. S. D. A., Brand G. D., Uehling D., Poda G., Krause H., Cummins C. L., Romeiro L. A. S. (2022). Phenolic Lipids
Derived from Cashew Nut Shell Liquid to Treat Metabolic Diseases. J. Med. Chem..

[46] Rossi M., Freschi M., De Camargo
Nascente L., Salerno A., De Melo
Viana Teixeira S., Nachon F., Chantegreil F., Soukup O., Prchal L., Malaguti M., Bergamini C., Bartolini M., Angeloni C., Hrelia S., Soares Romeiro L. A., Bolognesi M. L. (2021). Sustainable Drug Discovery of Multi-Target-Directed
Ligands for Alzheimer’s Disease. J. Med.
Chem..

[47] Parsons J. F., Calabrese K., Eisenstein E., Ladner J. E. (2003). Structure and Mechanism
of Pseudomonas Aeruginosa PhzD, an Isochorismatase from the Phenazine
Biosynthetic Pathway. Biochemistry.

[48] Grøftehauge M. K., Hajizadeh N. R., Swann M. J., Pohl E. (2015). Protein-Ligand Interactions
Investigated by Thermal Shift Assays (TSA) and Dual Polarization Interferometry
(DPI). Acta Crystallogr., Sect. D: Biol. Crystallogr..

[49] O’Toole G. A. (2011). Microtiter
Dish Biofilm Formation Assay. J. Vis.Exp..

[50] Schneider C. A., Rasband W. S., Eliceiri K. W. (2012). NIH Image to ImageJ: 25 Years of
Image Analysis. Nature Methods 2012 9:7.

[51] Cohen, J. Statistical Power Analysis for the Behavioral Sciences. Routledge: New York, 2013.

[52] Castillo-Juárez I., García-Contreras R., Velázquez-Guadarrama N., Soto-Hernández M., Martínez-Vázquez M. (2013). Amphypterygium
Adstringens Anacardic Acid Mixture Inhibits Quorum Sensing-Controlled
Virulence Factors of Chromobacterium Violaceum and Pseudomonas Aeruginosa. Arch Med. Res..

[53] Kanojia R. M., Murray W., Bernstein J., Fernandez J., Foleno B. D., Krause H., Lawrence L., Webb G., Barrett J. F. (1999). 6-Oxa Isosteres of Anacardic Acids
as Potent Inhibitors
of Bacterial Histidine Protein Kinase (HPK)-Mediated Two-Component
Regulatory Systems. Bioorg. Med. Chem. Lett..

[54] Cimmperman P., Baranauskiene L., Jachimovičiute S., Jachno J., Torresan J., Michailoviene V., Matuliene J., Sereikaite J., Bumelis V., Matulis D. (2008). A Quantitative Model
of Thermal Stabilization and Destabilization of Proteins by Ligands. Biophys. J..

[55] Muñoz-Cazares, N. ; García-Contreras, R. ; Pérez-López, M. ; Castillo-Juárez, I. Phenolic Compounds with Anti-Virulence Properties. In Phenolic Compounds—Biological Activity; InTech, 2017. 10.5772/66367.

[56] McGovern S. L., Caselli E., Grigorieff N., Shoichet B. K. (2002). A Common Mechanism
Underlying Promiscuous Inhibitors from Virtual and High-Throughput
Screening. J. Med. Chem..

[200] Bai N., Roder H., Dickson A., Karanicolas J. (2019). Isothermal
Analysis of ThermoFluor Data Can Readily
Provide Quantitative Binding Affinities. Sci.
Rep..

[57] Redhead M., Satchell R., Morkunaite V., Swift D., Petrauskas V., Golding E., Onions S., Matulis D., Unitt J. (2015). A Combinatorial
Biophysical Approach; FTSA and SPR for Identifying Small Molecule
Ligands and PAINs. Anal. Biochem..

[58] Xiao S., Tian H., Tao P. (2022). PASSer2.0:
Accurate Prediction of
Protein Allosteric Sites Through Automated Machine Learning. Front Mol. Biosci.

[59] Omage F. B., Salim J. A., Mazoni I., Yano I. H., Hernández
González J. E., Giachetto P. F., Tasic L., Arni R. K., Neshich G. (2025). STINGAllo: A Web Server for High-Throughput Prediction
of Allosteric Site-Forming Residues Using Internal Protein Nanoenvironment
Descriptors. Briefings Bioinf..

[60] Brenke R., Kozakov D., Chuang G.-Y., Beglov D., Hall D., Landon M. R., Mattos C., Vajda S. (2009). Fragment-Based Identification
of Druggable “hot Spots” of Proteins Using Fourier Domain
Correlation Techniques. BIOINFORMATICS ORIGINAL
PAPER.

[61] Sun Z., Wakefield A. E., Kolossvary I., Beglov D., Vajda S. (2020). Structure-Based
Analysis of Cryptic-Site Opening. Structure.

[62] Yueh C., Rettenmaier J., Xia B., Hall D. R., Alekseenko A., Porter K. A., Barkovich K., Keseru G., Whitty A., Wells J. A., Vajda S., Kozakov D. (2019). Kinase Atlas: Druggability
Analysis of Potential Allosteric Sites in Kinases. J. Med. Chem..

[63] Morais S. M., Silva K. A., Araujo H., Vieira I. G. P., Alves D. R., Fontenelle R. O. S., Silva A. M. S. (2017). Anacardic Acid Constituents from
Cashew Nut Shell Liquid: NMR Characterization and the Effect of Unsaturation
on Its Biological Activities. Pharmaceuticals
2017, Vol. 10, Page 31.

[64] Fu L., Shi S., Yi J., Wang N., He Y., Wu Z., Peng J., Deng Y., Wang W., Wu C., Lyu A., Zeng X., Zhao W., Hou T., Cao D. (2024). ADMETlab 3.0:
An Updated Comprehensive Online ADMET Prediction Platform Enhanced
with Broader Coverage, Improved Performance, API Functionality and
Decision Support. Nucleic Acids Res..

[65] Ohkawa I., Shiga S., Kageyama M. (1979). An Esterase on the Outer Membrane
of Pseudomonas Aeruginosa for the Hydrolysis of Long Chain Acyl Esters. J. Biochem.

[66] Rutherford, S. T. ; Bassler, B. L. Bacterial Quorum Sensing: Its Role in Virulence and Possibilities for Its Control. In Cold Spring Harbor Perspectives in Medicine. Cold Spring Harbor Laboratory Press, 2012. 10.1101/cshperspect.a012427 PMC3543102 23125205

[67] Huigens R.
W., Abouelhassan Y., Yang H. (2019). Phenazine Antibiotic-Inspired Discovery
of Bacterial Biofilm-Eradicating Agents. ChemBioChem..

[68] Jagani S., Chelikani R., Kim D. S. (2009). Effects of Phenol
and Natural Phenolic
Compounds on Biofilm Formation by Pseudomonas Aeruginosa. Biofouling.

[69] Ramos I., Dietrich L. E. P., Price-Whelan A., Newman D. K. (2010). Phenazines Affect
Biofilm Formation by Pseudomonas Aeruginosa in Similar Ways at Various
Scales. Res. Microbiol.

[70] Shrout J. D., Chopp D. L., Just C. L., Hentzer M., Givskov M., Parsek M. R. (2006). The Impact of Quorum
Sensing and Swarming Motility
on Pseudomonas Aeruginosa Biofilm Formation Is Nutritionally Conditional. Mol. Microbiol..

[71] de
la Fuente-Núñez C., Korolik V., Bains M., Nguyen U., Breidenstein E. B. M., Horsman S., Lewenza S., Burrows L., Hancock R. E. W. (2012). Inhibition of Bacterial Biofilm Formation
and Swarming Motility by a Small Synthetic Cationic Peptide. Antimicrob. Agents Chemother..

[72] Yeung A. T. Y., Torfs E. C. W., Jamshidi F., Bains M., Wiegand I., Hancock R. E. W., Overhage J. (2009). Swarming of
Pseudomonas Aeruginosa
Is Controlled by a Broad Spectrum of Transcriptional Regulators. Including MetR. J. Bacteriol.

[73] Butler M. T., Wang Q., Harshey R. M. (2010). Cell Density
and Mobility Protect
Swarming Bacteria against Antibiotics. Proc.
Natl. Acad. Sci. U. S. A..

[74] Kasallis S., Bru J. L., Chang R., Zhuo Q., Siryaporn A. (2023). Understanding
How Bacterial Collectives Organize on Surfaces by Tracking Surfactant
Flow. Curr. Opin Solid State Mater. Sci..

[75] Nozawa T., Tanikawa T., Hasegawa H., Takahashi C., Ando Y., Matsushita M., Nakagawa Y., Matsuyama T. (2007). Rhamnolipid-Dependent
Spreading Growth of Pseudomonas Aeruginosa on a High-Agar Medium:
Marked Enhancement under CO2-Rich Anaerobic Conditions. Microbiol. Immunol..

[76] Caiazza N. C., Shanks R. M. Q., O’Toole G. A. (2005). Rhamnolipids
Modulate Swarming Motility
Patterns of Pseudomonas Aeruginosa. J. Bacteriol..

[77] Dietrich L. E. P., Price-Whelan A., Petersen A., Whiteley M., Newman D. K. (2006). The Phenazine
Pyocyanin Is a Terminal Signalling Factor in the Quorum Sensing Network
of Pseudomonas Aeruginosa. Mol. Microbiol..

[78] Yang R., Guan Y., Zhou J., Sun B., Wang Z., Chen H., He Z., Jia A. (2018). Phytochemicals from
Camellia Nitidissima Chi Flowers Reduce the Pyocyanin Production and
Motility of Pseudomonas Aeruginosa PAO1. Front.
Microbiol..

[79] Bernabè G., Marzaro G., Di Pietra G., Otero A., Bellato M., Pauletto A., Scarpa M., Sut S., Chilin A., Dall’Acqua S., Brun P., Castagliuolo I. (2022). A Novel Phenolic
Derivative Inhibits AHL-Dependent Quorum Sensing Signaling in Pseudomonas
Aeruginosa. Front Pharmacol.

[80] Rütschlin S., Böttcher T. (2020). Inhibitors
of Bacterial Swarming Behavior. Chemistry
– A. European Journal.

[81] Zheng Y., Tsuji G., Opoku-Temeng C., Sintim H. O. (2016). Inhibition of P.
Aeruginosa c-Di-GMP Phosphodiesterase RocR and Swarming Motility by
a Benzoisothiazolinone Derivative. Chem. Sci..

[82] Furukawa S., Akiyoshi Y., O’Toole G. A., Ogihara H., Morinaga Y. (2010). Sugar Fatty
Acid Esters Inhibit Biofilm Formation by Food-Borne Pathogenic Bacteria. Int. J. Food Microbiol..

[83] Tremblay J., Richardson A. P., Lépine F., Déziel E. (2007). Self-Produced
Extracellular Stimuli Modulate the Pseudomonas Aeruginosa Swarming
Motility Behaviour. Environ. Microbiol.

[84] Fauvart M., Phillips P., Bachaspatimayum D., Verstraeten N., Fransaer J., Michiels J., Vermant J. (2012). Surface Tension Gradient
Control of Bacterial Swarming in Colonies of Pseudomonas Aeruginosa. Soft Matter.

[85] Winstanley C., O’Brien S., Brockhurst M. A. (2016). Pseudomonas Aeruginosa Evolutionary
Adaptation and Diversification in Cystic Fibrosis Chronic Lung Infections. Trends Microbiol.

[86] Bhagirath A. Y., Li Y., Somayajula D., Dadashi M., Badr S., Duan K. (2016). Cystic Fibrosis
Lung Environment and Pseudomonas Aeruginosa Infection. BMC Pulm Med.

[87] Poulsen B. E., Yang R., Clatworthy A. E., White T., Osmulski S. J., Li L., Penaranda C., Lander E. S., Shoresh N., Hung D. T. (2019). Defining
the core essential genome of Pseudomonas aeruginosa. Proceedings of the National Academy of Sciences.

