# Supplementary material for: Derivatives of the Cashew Nut Shell Liquid as Ligands of Pseudomonas aeruginosa Phenazine Protein D (PaPhzD)
Source: ACS Omega. 2025 Dec 15;10(51):63003–15. doi: 10.1021/acsomega.5c08941 (PMC12756723; doi:10.1021/acsomega.5c08941)
Supplement: Supplementary file 1 [file ao5c08941_si_001.pdf]

Derivatives of the cashew nut shell liquid as ligands of *Pseudomonas aeruginosa* phenazine protein D (*Pa*PhzD)

Authors: Marina Sena Mendes[#1], Thamires Quadros Froes[#2], Caio Gomes Tavares Rosa[4,5], Gabriella Simões Heyn Roth Cardoso[3], Thais Ferreira[3], Andressa Souza de Oliveira[3], Luiz A. S. Romeiro[3,4], Regina Lúcia Baldini[5], Marcelo S Castilho[1*]

[1] *Faculdade de Farmácia UFBA;*
[2] *Instituto de Pesquisa Gonçalo Moniz - Fiocruz, Bahia;*
[3]*Programa de Pós-graduação em Ciências Farmacêuticas, Faculdade de Ciências da Saúde, UnB;*
[4]*Programa de Pós-graduação em Medicina Tropical, Faculdade de Medicina, UnB;*
[5] *Departamento de Bioquímica, Instituto de Química, Universidade de São Paulo*

*Corresponding author; castilho@ufba.br; Faculdade de Farmácia, Universidade Federal da Bahia, 40.140-115 Salvador, Bahia, Brazil. Phone: +55 71 3283-6911

[#] Shared first authorship, as both authors contributed equally to this work

Table of Contents

Figure 1S: (A) Influence of different PaPhzD concentrations on the raw TSA fluorescence curve. (B) Effect of different DMSO concentrations over the *Pa*PhzD-unfolding transition (Tm). 3

Figure 2S: 1H RMN of LDT75 4

Figure 3S: 13C RMN of LDT75 4

Figure 4S: 1H RMN of LDT197 5

Figure 5S: 13C RMN of LDT197 5

Table 1S. Cross-reference between compound numbering and LDT codes 6

Computational studies 7

Figure 6S: Predicted allosteric regulation in PaPhzD (PDB ID 1NF9). (A) Allosteric site-forming residue (ASR) predicted by the StingAllo server (https://www.stingallo.cbi.cnptia.embrapa.br/sting). The protein is shown in gray cartoon, with the ASR (LEU120) highlighted in slate blue. Isochorismate (ISC) coordinates (PDB ID 1NF8) are shown to indicate the active site location in green stick. (B) Likelihood of allosteric pockets predicted by the Protein Allosteric Sites Server (https://passer.smu.edu/). Pocket locations are shown as spheres colored by their likelihood: yellow (87%), green (19.42%), cyan (17.37%), and magenta (17.18%). (C) Consensus sites (sticks – CS0 – CS9) were identified through in silico solvent mapping with Atlas software, when the active site is masked. (D) Consensus putative allosteric pocket (SAS 151.55 Å²) identified by all three in silico approaches. Basic residues (Arg41 and Arg105) within this pocket are highlighted in orange. ...8

Gene expression studies 9

Figure 7S: Effect of LDT10 (2) and LDT13 (3) on the phzA1-G1 (A) and phzA2-G2 transcription (B). 9

Cytotoxicity studies 10

Figure 8S: Cytotoxicity of compounds 3 and 4 against HEK293 and H9C2 cell lines. The compounds were tested at 25 µM, and cell viability was determined using the MTT assay. Cells under control conditions were treated with 1% DMSO (vehicle, v/v). The results are expressed as a percentage in relation to the control, taken as 100%. Filled bars represent HEK293 cells, and open bars represent H9C2 cells. Data are presented as mean values, and error bars indicate standard deviations from three independent experiments performed at least in triplicate....................10

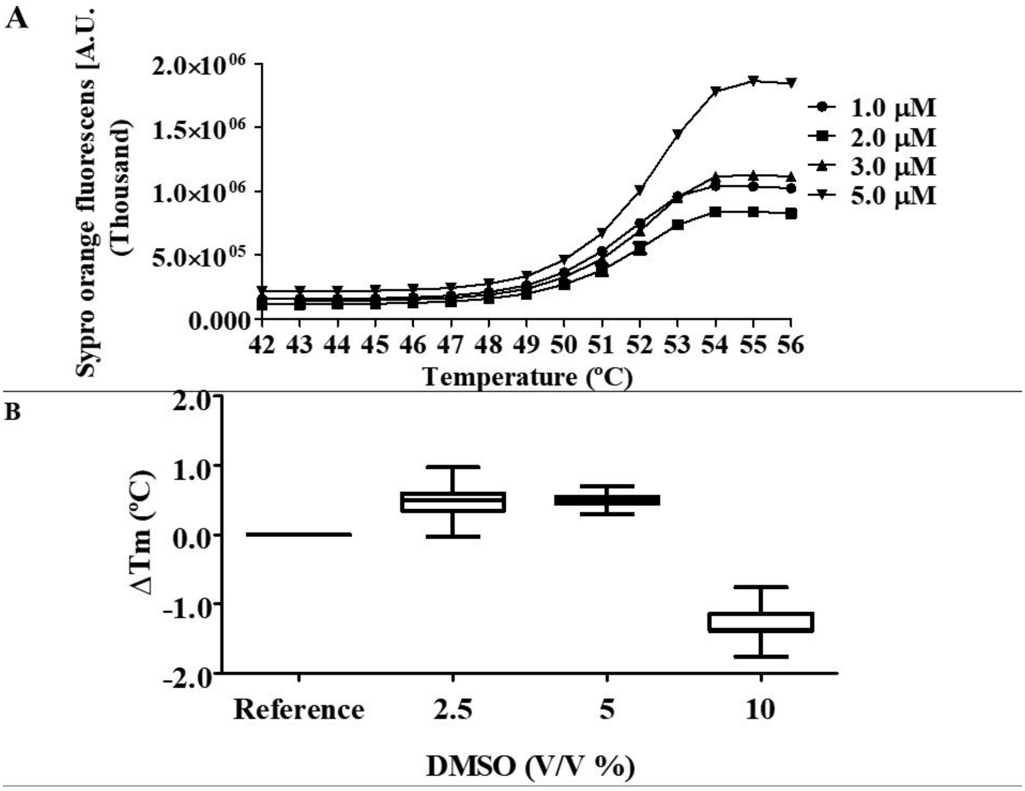

Figure 1S: (A) Influence of different *Pa*PhzD concentrations on the raw TSA fluorescence curve. (B) Effect of different DMSO concentrations over the *Pa*PhzD-unfolding transition (Tm).

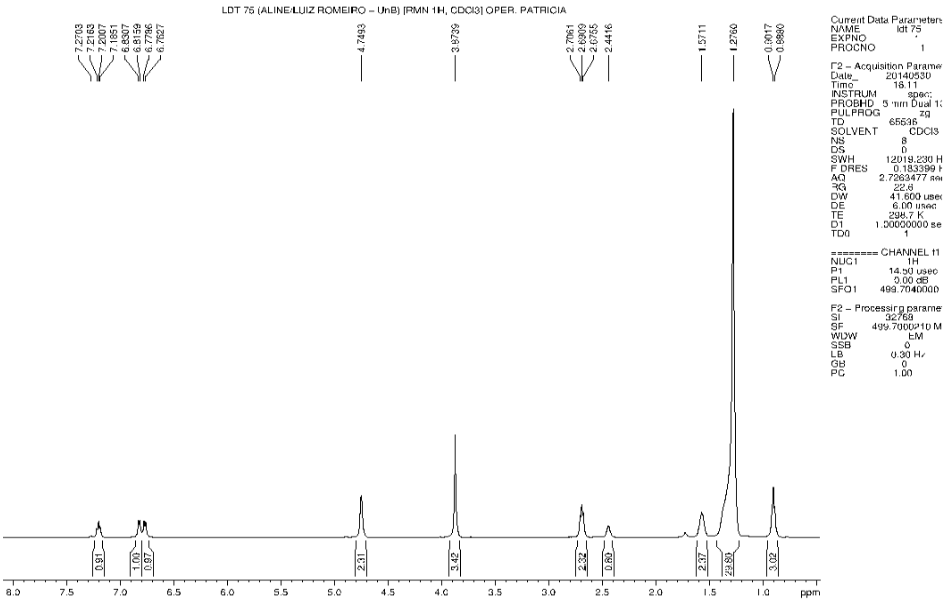

Figure 2S: 1H RMN of LDT75

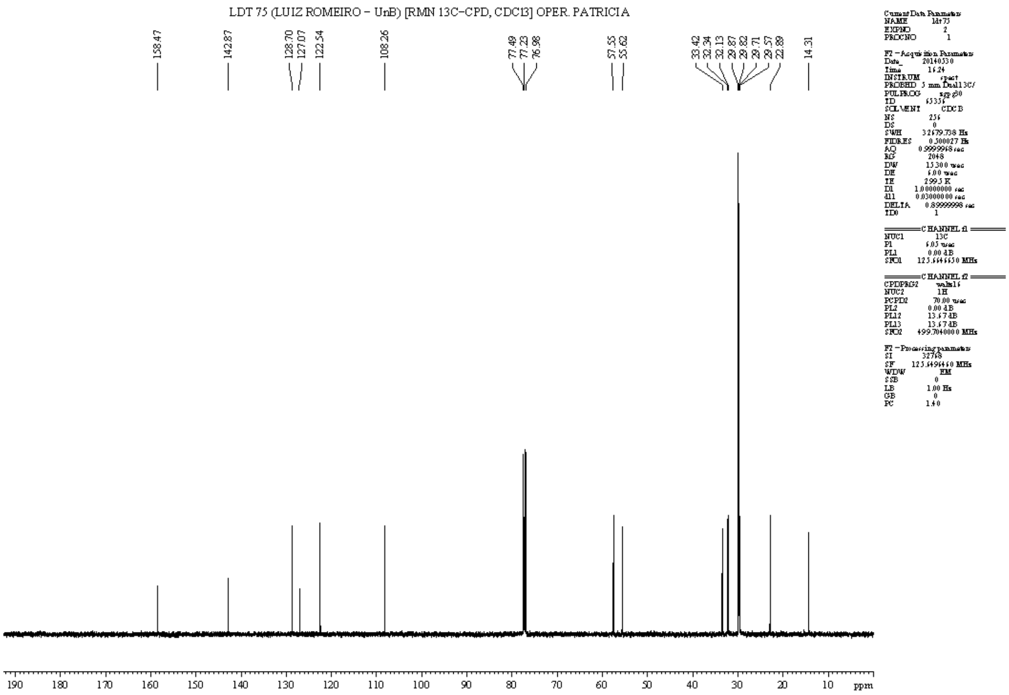

Figure 3S: 13C RMN of LDT75

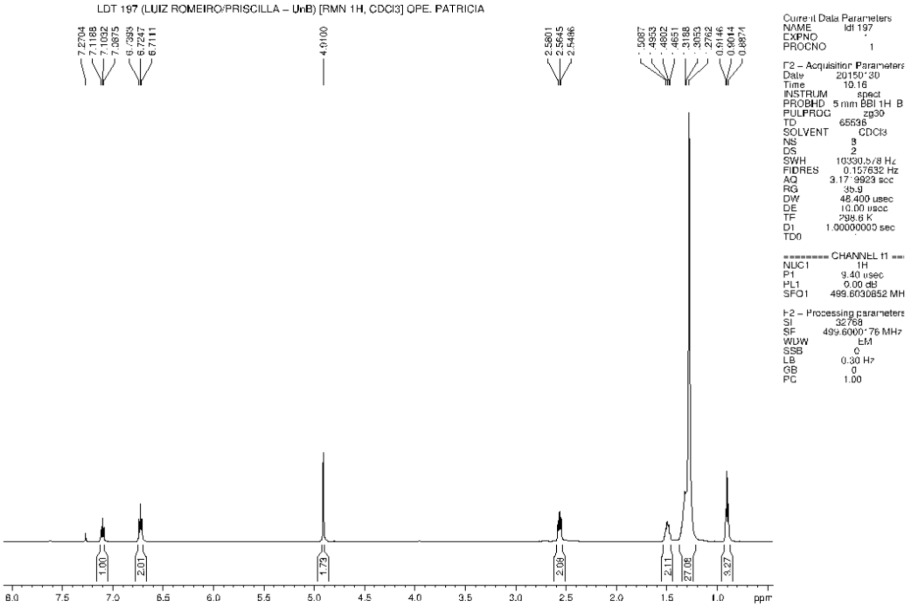

Figure 4S: 1H RMN of LDT197

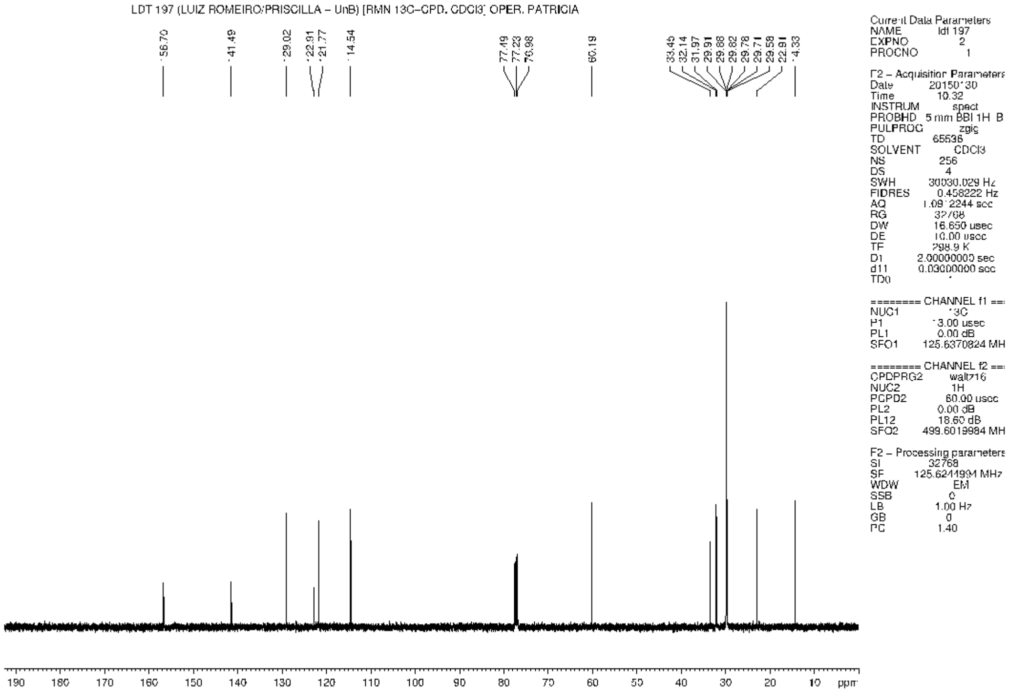

Figure 5S: 13C RMN of LDT197

Table 1S. Cross-reference between compound numbering and LDT codes

**1**	LDT11
**2**	LDT10
**3**	LDT13
**4**	LDT30
**5**	LDT75
**6**	LDT197
**7**	LDT29
**8**	LDT208
**9**	LDT28
**10**	LDT407
**11**	LDT12
**12**	LDT27
**13**	LDT15
**14**	LDT408
**15**	LDT16
**16**	LDT409
**17**	LDT77
**18**	LDT220

Computational studies

Allosteric Pocket Prediction

The crystallographic structure of *Pseudomonas aeruginosa* PhzD (PDB ID 1NF9, chain A) was analyzed using three online servers. First, the Protein Allosteric Sites Server (https://passer.smu.edu/) was used with default parameters (ensemble) for chain A (https://doi.org/10.3389/fmolb.2022.879251) to rank putative allosteric pockets likelihood. The same structure and chain were analyzed with the StingAllo server (https://www.stingallo.cbi.cnptia.embrapa.br/sting) to predict residues linked to allosteric regulation. The third approach employed solvent mapping using the Atlas software (https://acpharis.com/computational-solvent-mapping/), a stand-alone version of FTMap sharing the same algorithm to identify consensus binding sites for multiple organic solvents. To avoid detecting the orthosteric site, this active site was excluded during solvent mapping using the crystallographic coordinates of isochorismate (ISC) from the mutant PaPhzD structure (PDB ID 1NF8) (flags --box-pdb ISC --box-exclude). All results were visualized in PyMOL v2.7 (open-source) using scripts provided by each server.

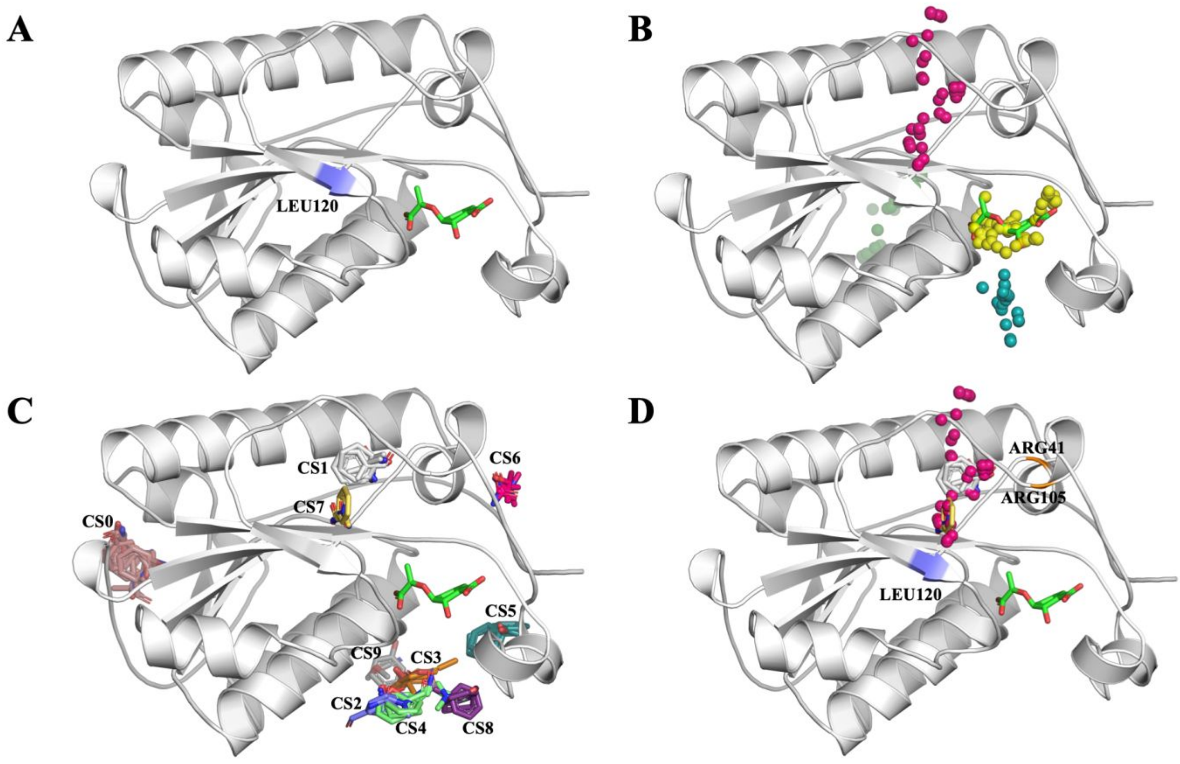

Figure 6S: Predicted allosteric regulation in *Pa*PhzD (PDB ID 1NF9). (A) Allosteric site-forming residue (ASR) predicted by the StingAllo server (https://www.stingallo.cbi.cnptia.embrapa.br/sting). The protein is shown in gray cartoon, with the ASR (LEU120) highlighted in slate blue. Isochorismate (ISC) coordinates (PDB ID 1NF8) are shown to indicate the active site location in green stick. (B) Likelihood of allosteric pockets predicted by the Protein Allosteric Sites Server (https://passer.smu.edu/). Pocket locations are shown as spheres colored by their likelihood: yellow (87%), green (19.42%), cyan (17.37%), and magenta (17.18%). (C) Consensus sites (sticks – CS0 – CS9) were identified through *in silico* solvent mapping with Atlas software, when the active site is masked. (D) Consensus putative allosteric pocket (SAS 151.55 Å^2) identified by all three *in silico* approaches. Basic residues (Arg41 and Arg105) within this pocket are highlighted in orange.

Gene expression studies

P. aeruginosa harbors two identically structured operons in its genome, which encode most of the enzymes responsible for PYO synthesis (*phzA1-G1* and *phzA2-G2*) but have slightly different regulatory regions [1]. *lacZ* transcriptional fusions for each operon were assayed at mid-log and late exponential phases (OD=1 and 3, respectively). At mid-log cultures, the expression of both QS-regulated operons is extremely low, and it increases more than 1000-fold at late exponential phase for *phzA1-G1* and 4-fold for *phzA2-G2*, as expected. Addition of compounds **2** and **4** had no effect for both operons in the late exponential phase, when the genes are expressed, suggesting that their effect in PYO synthesis is not related to inhibition at the transcriptional level.

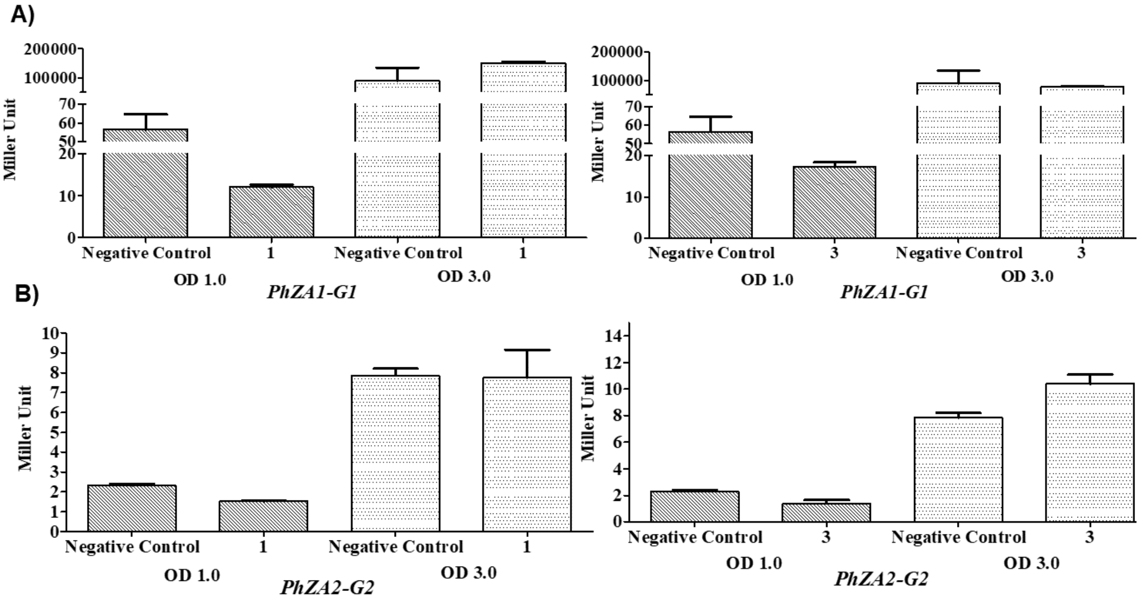

Figure 7S: Effect of LDT10 (2) and LDT13 (3) on the *phzA1-G1* (A) and *phzA2-G2* transcription (B).

Cytotoxicity studies

Cytotoxicity was evaluated in HEK293 and H9C2 cells using the colorimetric MTT assay (3-[4,5-dimethylthiazol-2-yl]-2,5-diphenyltetrazolium bromide), as described by Hansen et al [2]. The compounds were tested at a single concentration of 25 μM. MTT was added to each well at a final concentration of 2 mg/mL, and the cells were incubated for 2 h. Absorbance was measured at 570 nm using a microplate spectrophotometer. Cell viability was expressed as a percentage of the absorbance at 570 nm, with the control (1% DMSO, v/v) defined as 100%.

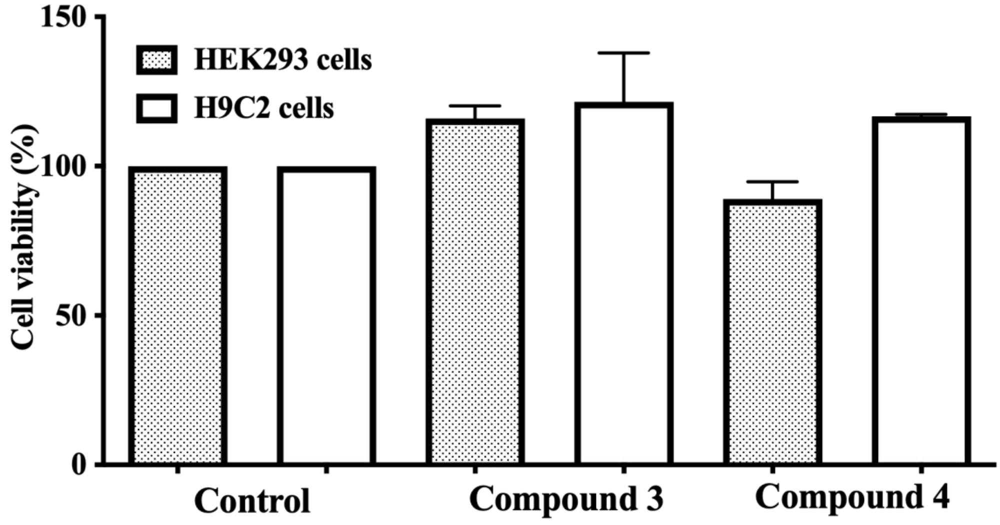

Figure 8S: Cytotoxicity of compounds 3 and 4 against HEK293 and H9C2 cell lines. The compounds were tested at 25 μM, and cell viability was determined using the MTT assay. Cells under control conditions were treated with 1% DMSO (vehicle, v/v). The results are expressed as a percentage in relation to the control, taken as 100%. Filled bars represent HEK293 cells, and open bars represent H9C2 cells. Data are presented as mean values, and error bars indicate standard deviations from three independent experiments performed at least in triplicate.

References

(1) Mavrodi, D. V; Bonsall, R. F.; Delaney, S. M.; Soule, M. J.; Phillips, G.; Thomashow, L. S. Functional Analysis of Genes for Biosynthesis of Pyocyanin

and Phenazine-1-Carboxamide from Pseudomonas Aeruginosa PAO1. *J Bacteriol* **2001**, *183* (21), 6454–6465. https://doi.org/10.1128/JB.183.21.6454-6465.2001.

(2) Hansen, M. B.; Nielsen, S. E.; Berg, K. Re-Examination and Further Development of a Precise and Rapid Dye Method for Measuring Cell Growth/Cell Kill. *J Immunol Methods* **1989**, *119* (2), 203–210. https://doi.org/10.1016/0022-1759(89)90397-9.
